# Functional Characterization of D9, a Novel Deazaneplanocin A (DZNep) Analog, in Targeting Acute Myeloid Leukemia (AML)

**DOI:** 10.1371/journal.pone.0122983

**Published:** 2015-04-30

**Authors:** Xia Jiang, Cheryl Zi Hui Lim, Zhimei Li, Puay Leng Lee, Siti Maryam J. M. Yatim, Peiyong Guan, Juntao Li, Jianbiao Zhou, Jingxuan Pan, Wee-Joo Chng, Christina L. L. Chai, Qiang Yu

**Affiliations:** 1 Cancer Therapeutics and Stratified Oncology, Genome Institute of Singapore, A*STAR (Agency for Science, Technology and Research), Biopolis, 138672, Singapore; 2 Computational and Systems Biology, Genome Institute of Singapore, A*STAR (Agency for Science, Technology and Research), Biopolis, 138672, Singapore; 3 Cancer Science Institute of Singapore, National University of Singapore, 14 Medical Drive, Singapore, 117599, Singapore; 4 Department of Pathophysiology, School of Medicine, Sun Yat-Sen University, 74 Zhongshan Road II, Guangzhou, 510089, China; 5 Department of Haematology-Oncology, National University Cancer Institute of Singapore, National University Health System, Singapore, Singapore; 6 Department of Medicine, Yong Loo Lin School of Medicine, National University of Singapore, Singapore, Singapore; 7 Institute of Chemical and Engineering Sciences, 8 Biomedical Grove, Neuros #07–01, Singapore, 138665, Singapore; 8 Department of Pharmacy, 18 Science Drive 4, National University of Singapore, Singapore 117543; 9 Cancer Institute, Jinan University, Guangzhou, 510632, China; 10 Department of Physiology, Yong Loo Lin School of Medicine, National University of Singapore, Singapore, 117597, Singapore; University of Navarra, SPAIN

## Abstract

Aberrant epigenetic events contribute to tumorigenesis of all human cancers. Significant efforts are underway in developing new generation of epigenetic cancer therapeutics. Although clinical trials for agents targeting DNA hypermethylation and histone deacetylation have yielded promising results, developing agents that target histone methylation remains to be in the early stage. We and others have previously reported that 3-Deazaneplanocin A (DZNep) is a histone methylation inhibitor that has a wide range of anticancer effects in various human cancers. Here, focusing on acute myeloid leukemia (AML) as a model, we reported a less toxic analog of DZNep, named D9, which is shown to be efficacious in AML cell lines and patient-derived samples in vitro, as well as AML tumorigenesis in vivo. Gene expression analysis in a panel of AML cell lines treated with D9 identified a set of genes that is associated with D9 sensitivity and implicated in multiple oncogenic signaling pathways. Moreover, we show that D9 is able to deplete the leukemia stem cells (LSC) and abolish chemotherapy-induced LSC enrichment, leading to dramatic elimination of AML cell survival. Thus, D9 appears to be a robust epigenetic compound that may constitute a potential for AML therapy.

## Introduction

Acute myeloid leukemia (AML) is an aggressive hematological disorder in which the haematopoietic progenitor cells lose their ability to differentiate normally and continue to proliferate. AML is an extremely heterogeneous disease with variable long term survival rate ranging from 20%-90% [1]). Although a number of targeted therapeutics have been proposed for treating AML, chemotherapy, such as cytarabine (Ara-C), adriamycin (ADR) or their combination, remains to be the first-line treatment option for most of the AML patients [2, 3]. In spite of an initial complete remission (CR) in nearly 70% of AML patients following the chemotherapy, a large portion of these patients subsequently relapse and eventually die of the disease progression [4]. It is generally thought that the disease recurrence stems from a rare subset of leukemia stem cells (LSCs) that are resistant to standard chemotherapy [5–8], which therefore raises a strong need to develop therapeutics to target LSCs.

Although there are growing interests in developing epigenetic therapy for hematological malignancies, the success for clinical advancement of histone deacetylase (HDAC) inhibitors and DNA methylation inhibitors remains to be limited for AML. Additionally, aberrant histone methylations, such as those induced by Polycomb protein Enhancer of Zeste homolog 2 (EZH2) [9, 10], Mixed-Lineage Leukemia (MLL) [11–13] and G9a [14–16] have been also shown to be attractive therapeutic targets. To date, efforts for developing histone methylation inhibitors are still in their infancy and no drugs have ever been approved by FDA or in late stages of clinical trials in AML and other malignancies.

We have previously reported that *S*-adenosylhomocysteine hydrolase inhibitor 3-deazaneplanocin A (DZNep) is a potent histone methylation inhibitor that is able to deplete the oncogenic PRC2 and associated histone H3 Lysine 27 trimethylation (H3K27me3) together with other histone methylations and induce robust apoptosis in cancer cells but not in normal cells [17]. Hereafter, there have been increasing numbers of reports showing the impressive anticancer effects of DZNep as a new epigenetic compound in a variety of cancer models both in vitro and in vivo [18–23]. In particular, DZNep alone, or in combination with HDAC inhibitor, has been shown to be effective in inducing growth arrest and apoptosis in AML cells [24, 25]. Therefore, there are interests in exploring DZNep and its analogs as a potential new class of epigenetic cancer therapeutics for AML.

In this study, we reported a comprehensive study of a novel analog of DZNep, named D9, in AML. We show that D9 induces strong growth inhibition and apoptosis in AML cells and its sensitivity in AML cells is associated with its ability to modulate gene expression associated with multiple oncogenic signaling pathways. Importantly, D9 is able to deplete both the basal and chemotherapy-enriched LSCs, and abrogate chemotherapy-induced gene expression associated with chemoresistance. Our results demonstrated a unique utility of D9 as a novel histone methylation inhibitor in targeting both bulk leukemic cells and leukemia stem cells (LSCs), thus providing a novel potential for eradication of AML.

## Materials and Methods

### Cell culture and drugs

A panel of acute myeloid leukemia (AML) cell lines, that is, MV4-11, Kasumi-1, KG-1, KG-1a, TF-1, TF-1a, MOLM-14, THP-1, HL-60 and Mono-Mac-1 was obtained as described previously [24]. D9 (MW: 308.16) was synthesized at Institute of Chemical and Engineering Sciences, Singapore. Suberoylanilide hydroxamic acid (SAHA), Adriamycin, Ara-C and Decitabine were purchased from Sigma-Aldrich.

### Patient samples

Frozen bone marrow (BM) blasts from newly diagnosed AML patients were obtained at the National University Hospital in Singapore with informed consent and approved by Institutional Review Board of National University Hospital. Written consents were obtained from the participants. Immediately after recovery, primary AML cells were cultured in IMDM supplemented with 10% FBS (Invitrogen), FLT3 ligand (20 ng/ml), SCF (20 ng/ml), IL-3 (20 ng/ml), G-CSF (50 ng/ml) and thrombopoietin (TPO; 50 ng/ml) (R&D Systems).

### Cell viability assay

Cells were seeded at a density of 1 × 10^3^ cells per well of 96-well optical bottom plate (Corning) 24 hours before D9 treatment. A range of concentrations of D9 diluted with basal RPMI or IMDM medium (Invitrogen) were added into the wells of cell suspension and incubated for 96 hours before detection. Six replicas were conducted for each sample. CellTiter-Glo Luminescent Cell Viability Assay (Promega) was used to evaluate the viable cell numbers. The luminescence signal was measured by MicroLumat Plus LB96V system (BERTHOLD TECHNOLOGIES). To calculate EC50, nonlinear regression sigmoidal dose response curves were generated using GraphPad PRISM3.

### Transwell migration and invasion assay

For invasion assay, 24-well Falcon FluoroBlok Transwell inserts (BD Biosciences) with a pore size of 8 μm were precoated with growth factor-reduced Matrigel (BD Biosciences) for 3–4 hours at 37°C before seeding the cells. For migration assay, the coating step was omitted. 5 × 10^4^ pretreated cells in 200 μl RPMI containing 0.25% FBS were seeded into the upper chamber of each insert. 750 μl RPMI containing 10% FBS were added outside the chamber as chemoattractant. Inserts were fixed after 24 hours of incubation at 37°C by using 3.7% formaldehyde for 20 minutes at room temperature. After several washes, the inserts were stained with 25 μg/ml propidium iodid for 30 minutes at room temperature in the dark. The migrated and invaded cells in 10 individual fields beneath the inserts were scanned and counted with Cellomics ArrayScan VTI (Thermo Scientific.). Triplicates were conducted for each sample, and at least three independent experiments were performed.

### Cell adhesion assay

96-well optical bottom plates were coated with 10 μg/ml Fibronectin (Sigma-Aldrich) for overnight at 4°C. The plates were washed three times using PBS prior to seeding the cells. 1 × 10^3^ TF-1a cells were seeded into each well and treated with D9 (100 nM) with or without Ara-C (50 nM). After 72 hours, the wells were gently washed by PBS three times to remove the unattached cells. CellTiter-Glo Luminescent Cell Viability Assay (Promega) was used for the quantification of the remaining adherent cells.

### Antibodies

The following primary antibodies were used. Cleaved PARP, EZH2, p-ERK (T202/204), p-AKT (S473), total ERK and total AKT (Cell Signaling Technology); Bim and Survivin (BD Biosciences); H3K27me3, H3K9me3, H3K4me3, H3K79me2, H4K20me3 and total H3 (Upstate Biotechnology); and actin (Sigma-Alrich) were used as primary antibodies. Anti-rabbit and anti-mouse horseradish peroxidise linked whole antibodies (GE Healthcare) were used as secondary antibodies.

### PI staining, active caspase3 staining and flow cytometric analysis

Treated cells were washed twice with cold PBS before fixing by 70% ethanol for at least 1 hour at 4°C. The fixed cells were washed twice with PBS, treated by 100 μl RNase (100 μg/ml) for 5 minutes, and then stained with 400 μl propidium iodide (PI) (50 μg/ml) for 30 minutes in the dark. The DNA contents were measured by FACSCalibur (Becton Dickson Instrument), and 1 × 10^4^ cells from each sample were analyzed by using Cell Quest software (Becton Dickinson Instrument). For active caspase3 assay, cytofix/cytoperm fixation/permeabilization kit (BD Biosciences) coupled with FITC rabbit anti active Caspase-3 antibody (BD Biosciences) were used to stain the cells for flow cytometric analysis.

### Immunofluorescent staining and flow cytometric analysis

TF-1a Cells were stained with CD38-FITC antibody and CD34-APC antibody (Miltenyi Biotec) sequentially. After staining, the cell pellets were suspended with 0.5 ml of 1 μg/ml propidium iodide (PI) just before harvested for analysis by FACSCalibur (BD Bioscience). Cell debris and dead cells were excluded from the analysis based on scatter signals and PI fluorescence. The stained cells were analyzed by using CellQuest software (BD bioscience).

### Microarray gene expression analyses and RT-PCR

Total RNA was isolated using the RNeasy Mini Kit (Qiagen). Microarray hybridization was performed using Human HT-12 V4.0 expression beadchip (Illumina), and data analysis was performed using GeneSpring GX (Agilent Technologies). The finalized genesets were imported into Ingenuity Pathway Analysis (IPA) (www.ingenuity.com) for gene ontology analysis. The microarray data reported herein have been deposited at the National Centre for Biotechnology Information’s Gene Expression Omnibus (accession no. GSE59624 and GSE59625). Reverse-transcription and quantitative PCR (qPCR) assays were performed using the High-Capacity cDNA Archive Kit and Kapa SyBr Fast qPCR Kit (Kapa Biosystems), respectively. Primer sequences are listed in S1 Table.

### Mouse xenografts and drug treatment

For subcutaneous implantation, 5 x 10^6^ MOLM-14 cells were subcutaneously injected into female athymic BALB/c nude mice (5–8 week-old). D9 at three different doses (30, 60 and 90 mg/kg) was administered though intraperitoneal (i.p.) injection as qd x 5d/w for 3 weeks in these recipients. Tumor diameters were measured every 3–4 days with calliper and the body weight of mice was monitored overtime. For bone marrow engraftment, 1 x 10^6^ MOLM-14 cells were transplanted into the sublethally irradiated NOD/SCID mice (5–8 week-old) mice through lateral tail vein injection. D9 (60 mg/kg) was administered through intraperitoneal (i.p.) injection two days after the tumor engraftment as q2d/w for 3 weeks. All animal studies were conducted in compliance with animal protocols approved by the A*STAR-Biopolis Institutional Animal Care and Use Committee (IACUC) of Singapore.

### Statistical analysis

Statistical analysis of this study was performed using GraphPad PRISM3. All values are expressed as mean ± SEM (standard error of mean) of at least three independent experiments. P values were calculated with the two-tailed Student’s t test. A p value < 0.05 is considered statistically significant.

## Results

### EC50 profiling of D9 in human cancer cell lines

**Fig 1:**
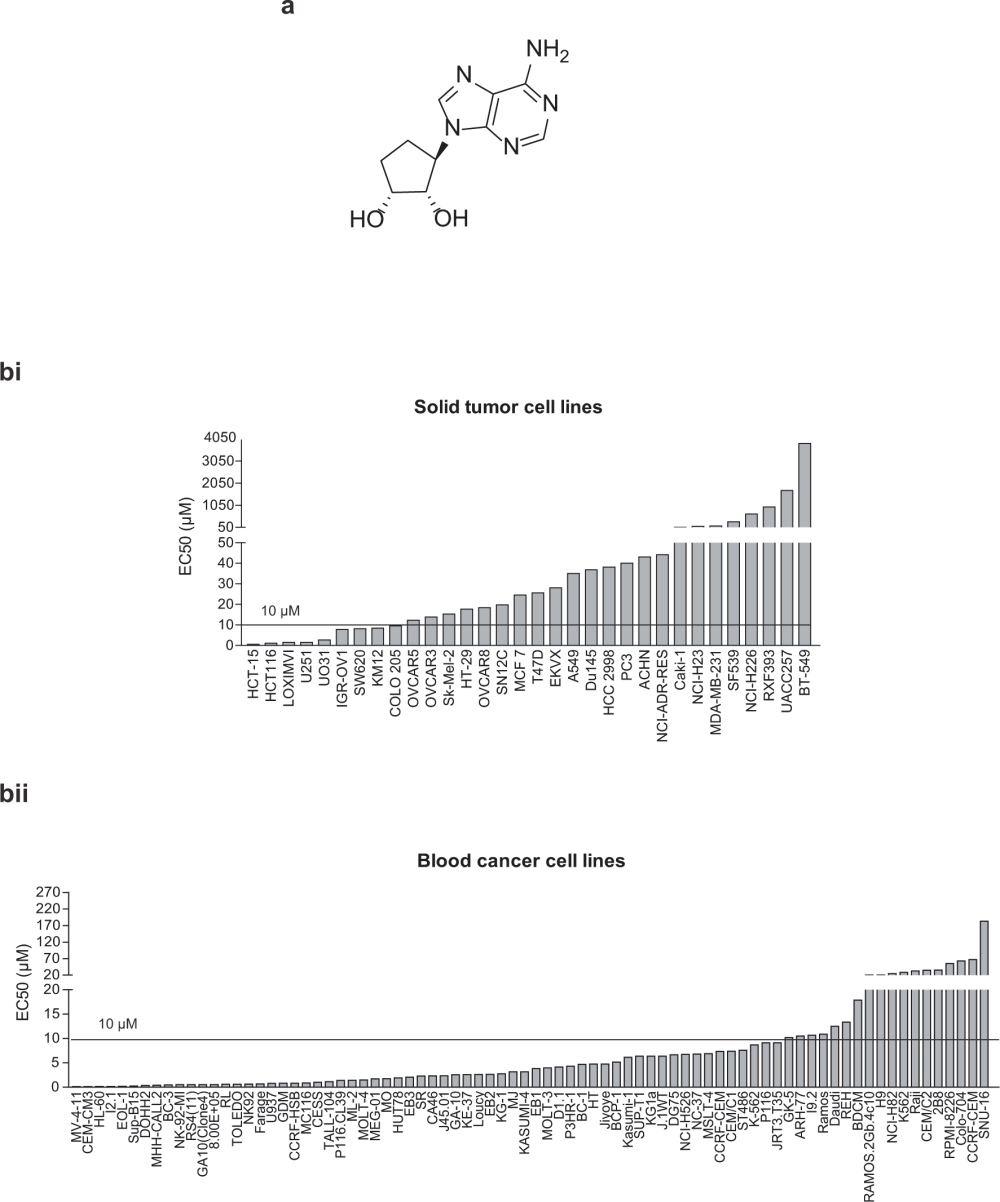
EC50 profile of D9 in human cancer cell lines. (a) Chemical structure of D9. (b) Bar graphs showing EC50 of D9 measured in a panel of solid (bi) and blood (bii) cancer cell lines using cell viability assay.1 x 10^^3^ cells of individual cell lines were seeded into 96-well plates in triplicates and D9 at 10 different doses was added 24 hours post cell seeding. Proliferation was measured after 96 hours treatment of D9 using an ATP based cell viability assay. EC50 of D9 was calculated by nonlinear regression (curve fit) using GraphPad PRISM3. Data represent the means of EC50 of D9 measured in three independent experiments, with each experiment run in triplicates.

In an effort to identify DZNep analogs with better potency and safety profile, we have synthesized a series of DZNep-like compounds and identified several lead compounds through structure and cellular activity relationship (SAR) analysis as well as correlation of chemical and physical properties. One such compound, D9 (Fig 1A), showed a comparable cellular activity with DZNep but a 20-fold less toxicity in mice [26] and was thus chosen for further investigations.

We first set out to determine which type of cancer cells will be most sensitive to D9. To do this, we screened a large panel of human cancer cell lines, including 32 solid tumor cell lines and 80 blood cancer cell lines, and determined their EC50 responses to D9. Overall, these cancer cell lines showed heterogeneous responses to D9; and blood cancer cell lines in general appeared to be more sensitive to D9 compared to solid tumor cell lines (median EC50 of 3.14 μM in blood cancer cell lines versus 25.1 μM in solid tumor cell lines) (Fig 1Bi and 1Bii; S2 and S3 Tables). Among the blood cancer cell lines, a significant number of AML cell lines such as MV4-11, HL-60, EOL1, and GDM-1 were highly sensitive to D9, with EC50s of < 1μM (Fig 1Bii). We thus decided to focus on AML for further investigation of the anti-cancer effect of D9.

### In vitro activity of D9 in AML cell lines

**Fig 2:**
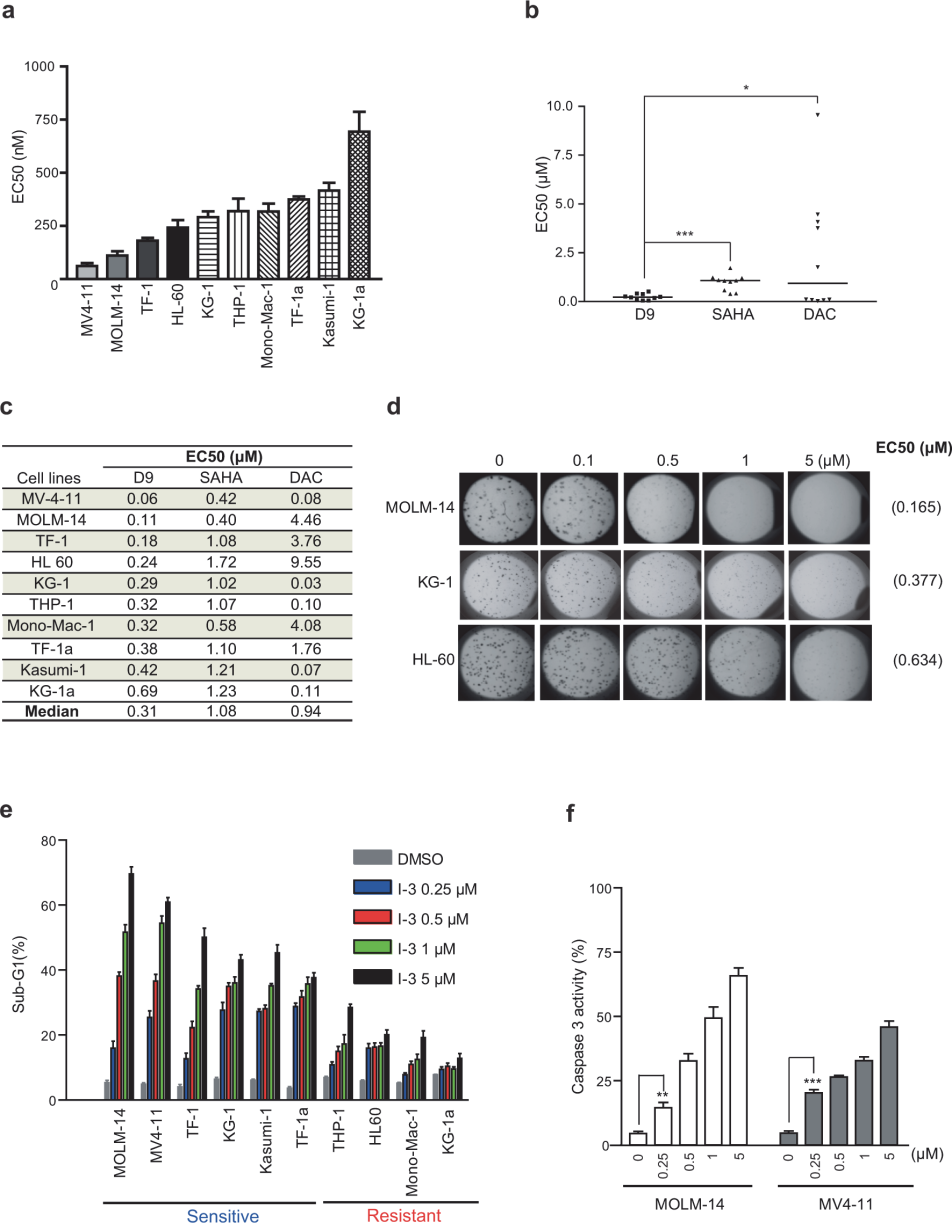
In vitro anti-leukemia activity of D9 in AML cell lines. (a) Bar graph showing EC50 of D9 measured in a panel of AML cell lines using cell viability assay. (b) Dot plot showing EC50 of D9, SAHA and DAC measured in the same panel of AML cell lines. The short lines represent the median EC50 of each compound in the AML cell line panel. (c) Table showing EC50 of D9, SAHA and DAC measured in the AML cell line panel. (d) Representative images of soft agar assay conducted in MOLM-14, KG-1 and HL-60 cells. 1 x 10^^3^ cells were mixed with D9 at various doses in semi-solid media and incubated for 2 weeks. Colony growth was assessed and EC50 of D9 measured by soft agar assay was indicated in the brackets. Data represent the means of EC50 of D9 measured in three independent experiments, with each experiment run in triplicates. (e) Bar graphs showing sub-G1 percentage of 10 AML cell lines treated with indicated concentrations of D9 for 72 hours, followed by propidium iodide (PI) staining and fluorescence-activated cell sorting (FACS) analysis. (f) Bar graphs showing the percentage of cells positive for active Caspase 3 in MOLM-14 and MV4-11 cells treated with indicated concentrations of D9 for 72 hours, followed by active Caspase 3 antibody staining and FACS analysis. Data are mean ± SEM; N = 3; *P < 0.05, **P < 0.01, ***P < 0.001, unpaired two tailed t test.

To investigate the effect of D9 on AML, we further validated the effect of D9 on the short-term viability of a panel of 10 well-established AML cell lines. For comparison, we also included histone deacetylase (HDAC) inhibitor SAHA and DNA methylation inhibitor Decitabine, both of which are FDA approved for the treatment of hematological malignancies [27, 28]. The results showed that in general AML cell lines were remarkably more sensitive to D9 compared to either SAHA or DAC, with a median EC50 of 0.31 μM for D9, 1.08 μM for SAHA and 0.94 μM for DAC, respectively (Fig 2B and 2C), indicating that D9 is potentially a more potent compound in AML cell lines compared to SAHA and DAC. Moreover, in a 14-day long soft agar colony formation assay, D9 was also effective in reducing the colony growth of MOLM-14, KG-1 and HL-60 cells in a dose-dependent manner, with similar EC50s as determined by the short-term cell viability assay (Fig 2D). Finally, we show that D9 was able to induce a sub-G1 cell death in MV4-11, MOLM-14, TF-1, KG-1, Kasumi-1 and TF-1a cells (45–75%), but to lesser extents in THP-1, HL-60, Mono-Mac-1and KG-1a cells (< 20%) (Fig 2E). The D9-induced cell death was further confirmed to be apoptosis by measuring Caspase 3 activation in MOLM-14 and MV4-11 cells (Fig 2F).

### Effects of D9 on histone methylation and transcriptome changes associated with D9-induced apoptosis in AML cells

**Fig 3:**
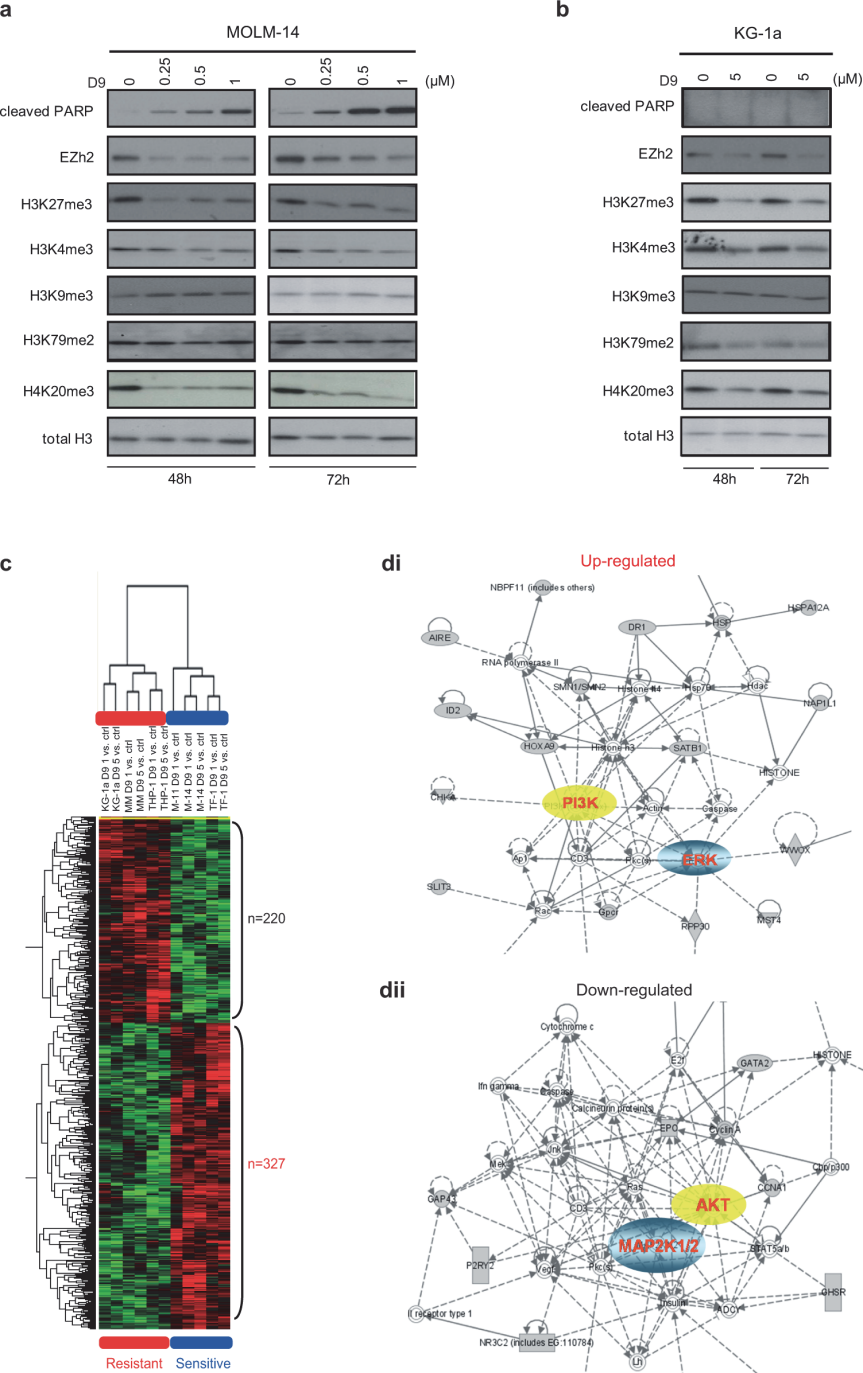
Effects of D9 on histone methylation and transcriptome in AML. Western blot analysis showing the level of cleaved PARP, EZh2 and a series of histone lysine methylation marks in MOLM-14 (a) and KG-1a cells (b) treated by D9 at indicated concentrations for 48 and 72 hours. (c) Heat map of differential genesets between sensitive and resistant cell lines with D9 treatment. Three sensitive (MOLM-14, MV4-11 and TF-1) and three resistant cell lines (Mono-Mac-1, KG-1a and THP-1) were treated with D9 at 1 or 5 μM for 48 hours. Total RNA was isolated for microarray and SAM analysis. 327 genes were up-regulated and 220 genes were down-regulated upon D9 treatment in sensitive cells relative to resistant cells using 10% false discovery rate (FDR) cut-off. (d) Ingenuity Pathway Analysis (IPA) of differentially up-regulated geneset and down-regulated geneset (dii) showing their strong connections to PI3K/AKT and MEK/ERK signaling pathways.

We next evaluated the overall effects of D9 on histone lysine methylations in sensitive MOLM-14 and resistant KG-1a to determine if D9 would have differential effects on the histone methylations in the two cell lines. In both cell lines we showed that D9 induced similar levels of suppression of H3K27me3 and H4K20me3 and to lesser extents on H3K4me3 and H3K79me2, while it had little effects on H3K9me2 and H3K9me3 (Fig 3A and 3B). This observation is similar to the parental compound DZNep that has been shown to act as a selective but non-specific histone methylation inhibitor in cancer cells, including AML [17, 24]. Thus, the differential sensitivities of AML cells to D9 do not seem to be associated with its ability to inhibit the bulk histone methylation per se, but perhaps more likely to be associated with the differential gene expression response to D9.

To test this hypothesis, we treated three sensitive (MOLM-14, MV4-11 and TF-1) and three resistant (Mono-Mac-1, THP-1, and KG-1a) cell lines with D9 for 48 hours and performed Illumina BeadChip transcriptom profiling analysis. Significance Analysis of Microarrays (SAM) analysis shows that D9 treatment resulted in transcriptional changes of 547 genes, including 327 upregulated genes and 220 downregulated genes in sensitive cell lines but not in resistant cell lines, as shown in a heatmap of supervised gene clustering (Fig 3C; S4 and S5 Tables). Ingenuity pathway analysis (IPA) of both gene sets revealed gene networks linked to the PI3K/AKT and MEK/ERK pathways (Fig 3Di and 3Dii), whose aberrant activation is frequently observed in AML [29, 30]. This analysis raised the possibility that D9 treatment induced a transcriptional change that may result in modulations of PI3K/AKT and MAPK signaling cascades in D9-sensitive cell lines but not resistant cell lines.

### D9 treatment suppresses PI3K and MAPK signaling in both AML cell lines and primary patient samples

**Fig 4:**
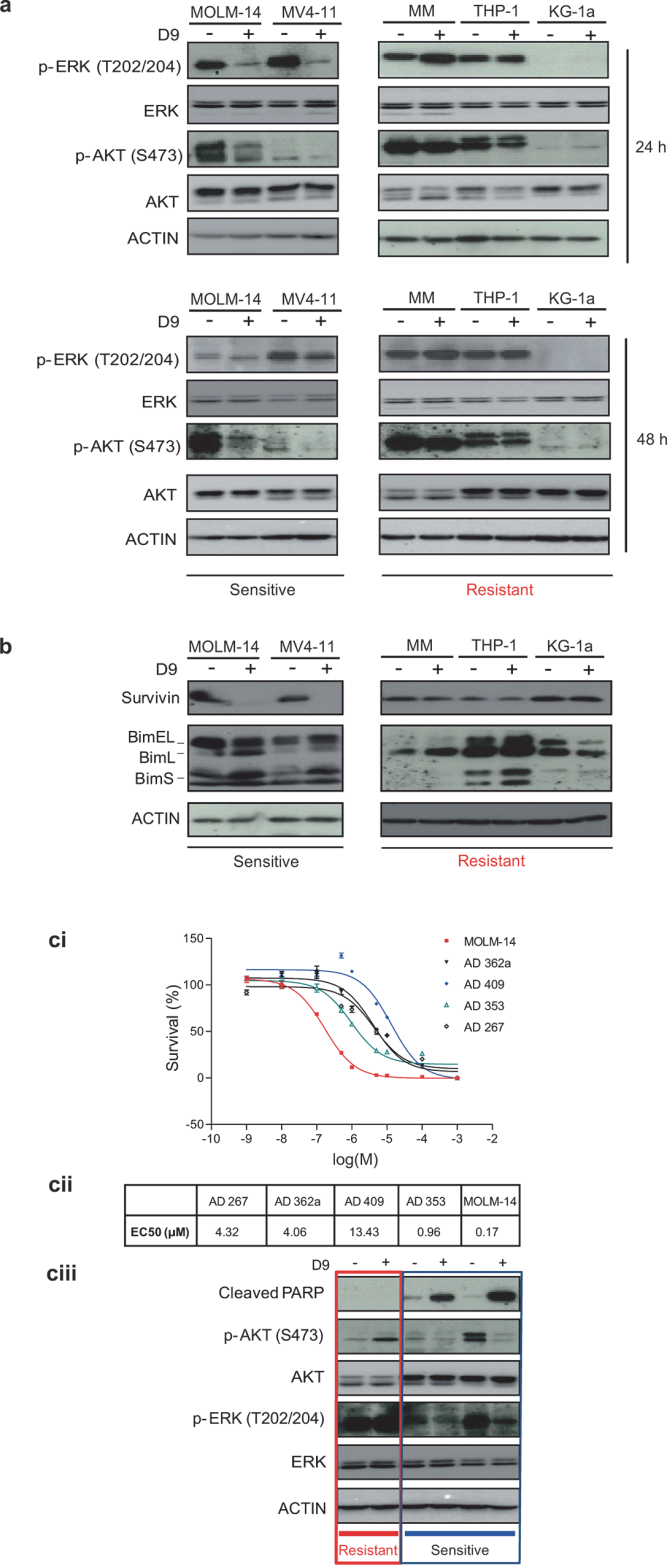
Effects of D9 on AKT and ERK phosphorylation in AML. (a)Western blot analysis of p-ERK (T202/204), total ERK, p-AKT (S473), total AKT and ACTIN in indicated AML cell lines treated with D9 for 24 hours and 48 hours. (b) Western blot analysis showing the effects of D9 on Bim and Survivin in AML cell lines as in (a). (c) The frozen primary AML patient blasts were recovered for 24 hours and the dead cells were removed using Dead Cell Removal Kit just before D9 treatment. The blasts were treated for 96 hours for cell viability assay and 48 hours for western blot analysis. The diagrams showed the drug response curves of AML patient blasts towards D9 (ci), measurement of EC50 of D9 using cell viability assay (cii) and western blot analysis of PARP, p-ERK (T202/204), total ERK, p-AKT (S473), total AKT and ACTIN in AML patient blasts as well as MOLM-14 with and without D9 treatment (ciii). Each data point in the plots of drug response curves of D9 represents the mean ± SEM of six replicates at each specified concentration of D9, N = 3.

To validate the above hypothesis, we investigated the effects of D9 on the PI3K and MAPK pathways by western blot analysis in two most sensitive cell lines MOLM-14 and MV4-11 and three resistant cell lines Mono-Mac-1 (MM), THP-1 and KG-1a. Consistent with what we have predicted from the gene expression analysis, D9 treatment resulted in significant suppression of MAPK and PI3K signaling as indicated by reduced phosphorylation of ERK (T202/204) or AKT (S473) in sensitive MOLM-14 and MV4-11 cells at both 24 and 48 hours without affecting the total levels of either ERK or AKT (Fig 4A). In contrast, the three resistant cells did not show such changes on p-ERK (T202/204) or p-AKT (S473) (Fig 4A).

It is well known that ERK inhibition results in upregulation of pro-apoptotic Bim induction [31, 32]. Accordingly, we found that all the three Bim isoforms, in particular BimS, the most potent isoform for apoptosis [33], were upregulated by D9 in sensitive lines but not in resistant lines. On the other hand, expression of the anti-apoptotic molecule, Survivin, which is a downstream effector of AKT [34, 35], was suppressed by D9 only in the sensitive cell lines (Fig 4B). Thus, the combined changes in Bim and Survivin induced by D9 are consistent with the strong apoptosis observed in the sensitive cell lines. Based on these observations, we proposed that the molecular mechanisms underlying the D9 sensitivity in AML cells is associated with a transcriptomic response resulting in downregulation of oncogenic PI3K-AKT and MAPK signaling pathways, leading to apoptosis.

To validate the clinical relevance of these findings, we investigated whether D9 has a similar effect on patient-derived primary AML cells. To this end, we managed to test the effect of D9 on 4 sets of primary AML blasts isolated from four newly diagnosed AML patients. The result shows that the primary blasts, like AML cell lines, exhibited heterogeneous responses to D9, with EC50 of D9 ranging from 0.96 to 13.43 μM (Fig 4Cii and S6 Table). In the sensitive patient sample AD353, we detected a robust PARP cleavage and strong reductions of phosphorylated AKT and ERK, which was similar to MOLM-14 line included here as a positive control. In contrast, we did not see such changes in resistant AD409 sample. In fact, we even observed a slight activation of AKT and ERK signaling in AD409 (Fig 4Ciii), probably a result of a feedback activation. Thus, D9 is able to target multiple oncogenic signaling pathways and this effect seems to be associated with the therapeutic effect in sensitive AML cells sourced from both the cell lines and patient-derived primary cells.

### D9 inhibits AML tumorigenesis in vivo

**Fig 5:**
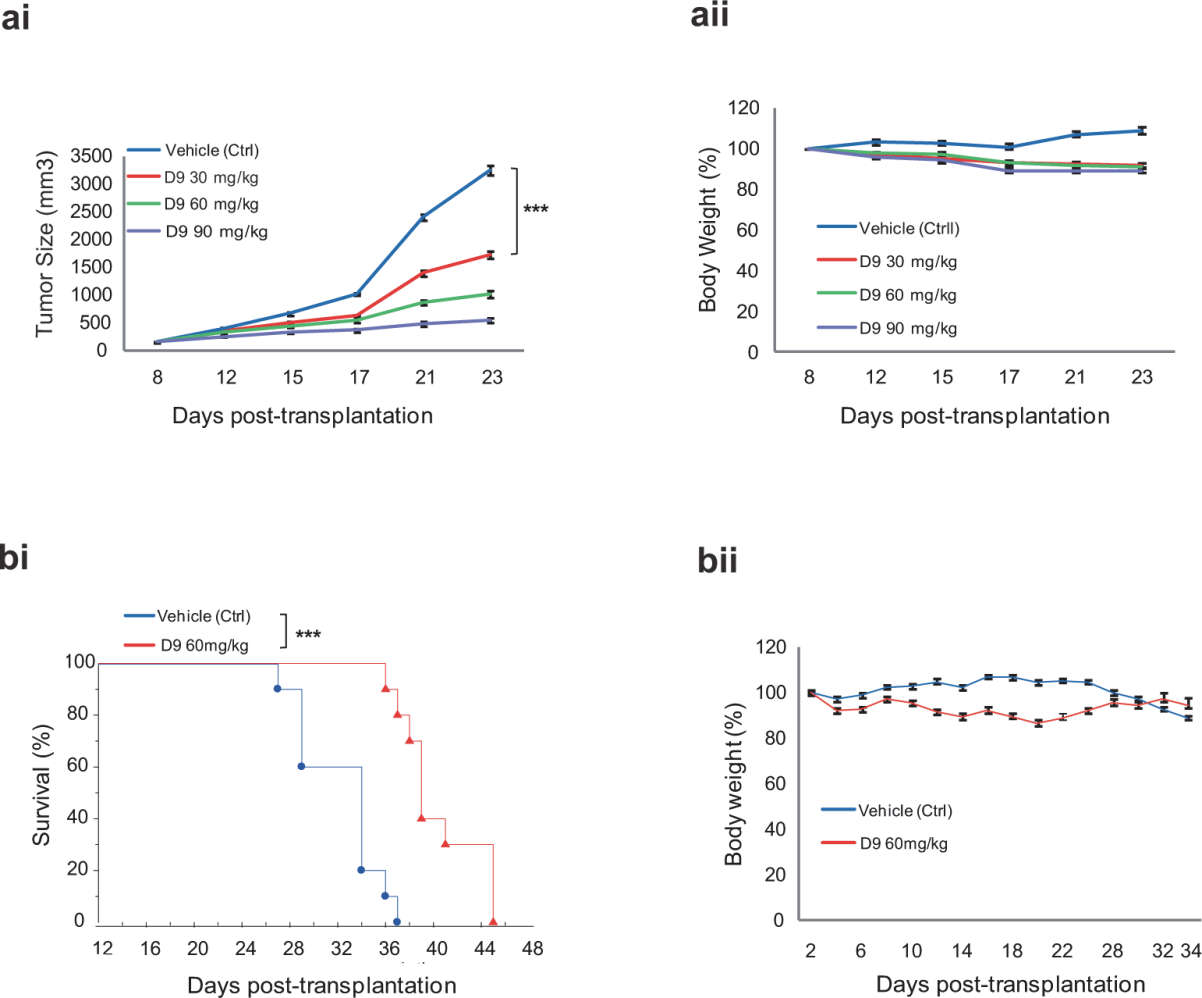
In vivo effects of D9 in AML mouse models. (a) Subcutaneous xenograft tumor growth of MOLM-14 cells in Balb/c mice treated with D9. 5 x 10^6 of MOLM-14 cells were injected into mice subcutaneously (s.c.). D9 at three different doses (30, 60 and 90 mg/kg) was administered through intraperitoneal (i.p.) injection as qd x 5d/w for 3 weeks in these recipients from day 8 to day 23 after transplantation, with vehicle treatment as control. The graphs show the tumor sizes (ai) and body weight change (aii). Data are mean ± SEM, ***P < 0.001, unpaired two tailed t test. (b) Kaplan–Meier curves of NOD/SCID mice treated with D9. 1 x 10^6 of MOLM-14 cells were injected into sublethally irradiated NOD/SCID mice through tail vein. D9 at 60 mg/kg was administered through i.p. injections as q2d/w for 3 weeks in these recipients from day 2 after transplantation, with vehicle treatment as control. The graphs showed the survival rate (bi) and body weight change (bii) during D9 treatment. Data are mean ± SEM, ***P <0.001, log-rank (Mantel-Cox) test conducted.

To evaluate the therapeutic potential of D9 in vivo, we used both the xenograft tumor growth model and bone marrow engraftment model. First, in a model of MOLM-14-derived subcutaneous transplantation in nude mice, D9 administered daily at 30, 60 and 90 mg/kg though intraperitoneal (i.p.) injection resulted in marked inhibition of tumor growth in a dose-dependent manner (Fig 5Ai). The treatment did not cause overt signs of toxicity as judged by insignificant body weight loss of mice before and after the D9 treatment (Fig 5Aii), suggesting that the effective doses of D9 were well tolerated in the recipient mice.

We next determined the anticancer efficacy of D9 in a more therapeutically relevant model of AML, in which MOLM-14 cells were injected through the tail vein of sublethally irradiated NOD/SCID mice to induce leukemic dissemination and mortality. We found that D9 given at 60 mg/kg through i.p. injection caused dramatic reduction in leukemic burden (data not shown) and significant prolonged life span of recipient mice (median survivals of 42 days in D9 group vs. 36 days in control group, p < 0.001) (Fig 5Bi). Again, D9 in this model was also well tolerated and no overt toxicity was observed in recipient mice (Fig 5Bii). Collectively, our results showed that D9 possessed potent anti-AML effects both in vitro and in vivo.

### D9 treatment depletes both basal and chemotherapy-induced LSC

**Fig 6:**
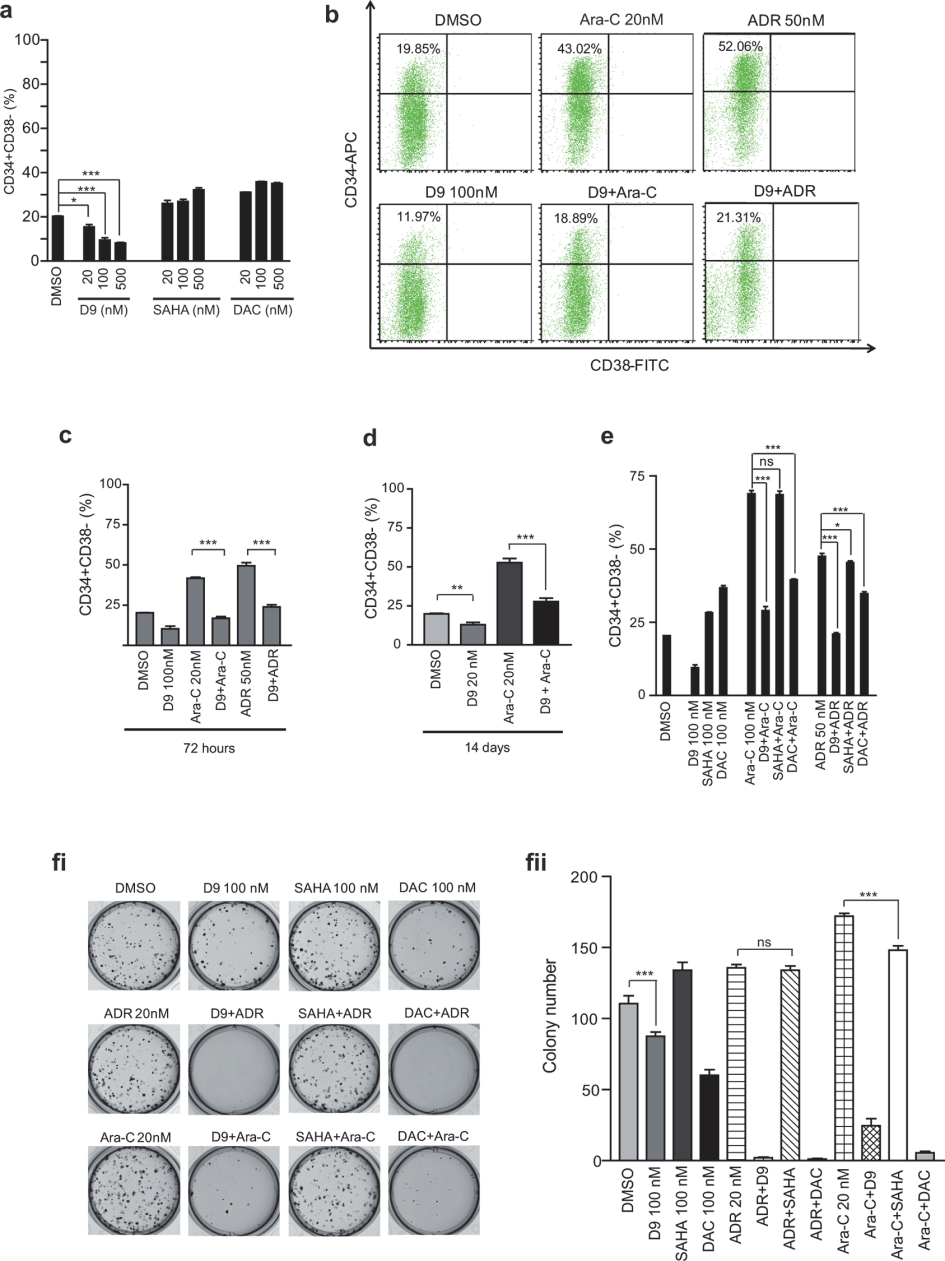
Effective anti-leukemia stem cells (LSC) activity of D9 in AML. (a) Bar graphs showing FACS analysis of the proportion of CD34+CD38- population in TF-1a after the single treatment with D9, SAHA or DAC for 72 hours. (b) Representative FACS histogram profiles of CD34+CD38- cell population in TF-1a cells treated with D9 (100 nM) alone or in combination with either Ara-C (20 nM) or ADR (50 nM). (c) Bar graphs showing the proportion of CD34+CD38- population in TF-1a cells treated as in B. (d) Bar graphs showing the proportion of CD34+CD38- population in TF-1a treated with D9 (20 nM) with or without Ara-C (20 nM) for 14 days. (e). Bar graphs showing the percentage of CD34+CD38- cell population in TF-1a cells treated as indicated. (f) Colony Formation Unit (CFU) assay showing the effects of D9, SAHA or DAC on basal or Ara-C or ADR-induced colony formation capacity of TF-1a cells. The medium and drugs were replenished every 3 to 4 days and the dead cells were removed by Dead Cell Removal Kit. 1 x 10^3 live cells were seeded with semi-solid colony formation medium and incubated for 2 weeks before enumeration. Representative images of the colony formation of TF-1a were shown on (fi) and bar graphs were shown the colony numbers on (fii). Data are mean ± SEM; N = 3; *P < 0.05, **P < 0.01, ***P < 0.001, ns represents no significance, unpaired two tailed t test.

Because tumor initiating cells or cancer stem cells have been thought to have undergone epigenetic reprogramming [36, 37], we sought to determine if D9 as a histone methylation inhibitor would be effective in targeting AML leukemia stem cells (LSCs). To test the effect of D9 on LSCs, we used TF-la, an AML primitive myeloid progenitor cell line, which has a high fraction of stem-like CD34+CD38- cells and thus considered as an excellent model for studying LSC [38]. Treatment of TF-1a cells with increasing low nanomolar concentration of D9 for 72 hours resulted in efficient depletion of CD34+CD38- population (Fig 6A). For a comparison, we also included HDAC inhibitor SAHA and DNA methylation inhibitor DAC in this experiment. Interestingly, we did not observe such an effect for SAHA and DAC (Fig 6B).

Chemotherapy is well known to induce enrichment of cancer stem cell, which is believed to cause chemoresistance and disease recurrence. We next tested if D9 is able to reduce chemotherapy-induced LSC enrichment. As expected, both Ara-C and ADR treatment of TF-1a cells for 72 hours resulted in dramatic increases of CD34+CD38- populations, while co-treatment with D9 (100 nM) nearly completely abrogated such increases (Fig 6B and 6C). Moreover, in a 14 days long-term experiment, D9 used even at 20 nM was also able to sufficiently deplete the CD34+CD38- enrichment by Ara-C (Fig 6D). Again, SAHA did not show such an effect to antagonize the chemotherapy-induced LSC enrichment, while DAC showed a much weaker effect than D9 in targeting LSC (Fig 6E).

To further evaluate the effects of D9 in the context of LSC survival associated with chemoresistance, we used a colony-forming-unit (CFU) assay, a gold standard in vitro assay to evaluate a long term survival of haematopoietic or leukemia stem/progenitor cells. In this experiment, TF-1a cells were treated with Ara-C or ADR, and then the dead cells were removed by using Dead Cell Removal Kit and the remaining live cells were seeded with equal numbers for CFU assay. Consistent with the induction of LSC by chemotherapy, we observed more colonies formed in Ara-C- or ADR-treated cells compared to untreated control (Fig 6F). Strikingly, co-treatment with D9, but not SAHA, nearly completely abolished the colony formation induced by Ara-C or ADR (Fig 6F). Under the same condition, DAC exerted comparable inhibitory effect on the colony formation of TF-1a cells (Fig 6F). These findings further suggest that D9 as a histone methylation inhibitor is equipped with a special ability to remove LSC and thus the chemoresistance in AML.

### D9 abolishes chemotherapy-induced gene expression associated with drug resistance in AML

**Fig 7:**
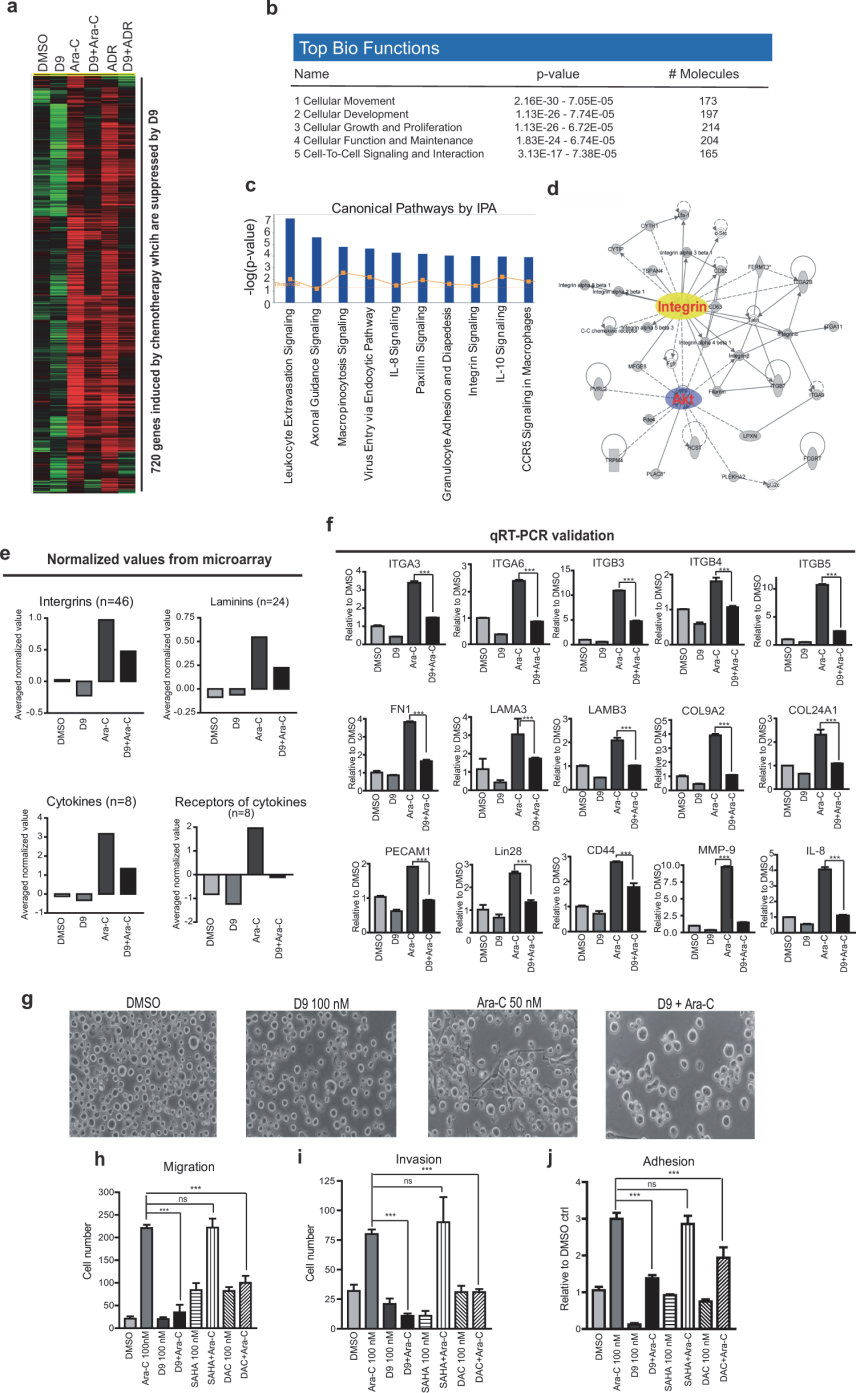
D9 targets cell adhesion-mediated drug resistance (CAM-DR) in AML. (a) Heat map of 720 genesets induced by both Ara-C and ADR which were suppressed by D9. (b) The diagram showing the top five Bio Functions of commonly upregulated 720 genes by Ara-C and ADR suggested by IPA. (c) The diagram showing the top ten canonical pathways of commonly upregulated 720 genes by Ara-C and ADR suggested by IPA. (d) The diagrams showing Integrins and AKT are highly associated networks of commonly upregulated 720 genes by Ara-C and ADR suggested by IPA. (e) Bar graphs showing the averaged values of 46 probes of Integrin members, 24 probes of Laminins, 8 probes of cytokines and 8 probes of receptors of cytokines extracted from normalized microarray data of CD34+CD38- double-selected TF-1a cells treated as in A. (f) Bar graphs showing qRT-PCR validation of microarray expression data of 15 representative genes as indicated. (g) Representative phase-contrast images of TF-1a cells treated with D9 (100 nM), Ara-C (50 nM) alone or combination. Bar graphs showing transwell migration assay (h), invasion assays (i) and adhesion assay (j) conducted on TF-1a cells treated with D9 (100 nM), SAHA (100 nM) or DAC (100 nM) with or without Ara-C (100 nM). Data are mean ± SEM; N = 3; *P < 0.05, **P < 0.01, ***P < 0.001, ns represents no significance, unpaired two tailed t test.

We next sought to characterize the molecular changes induced by the chemotherapy that is antagonized by D9. To do this, TF-1a cells were treated with Ara-C or ADR or co-treated with D9 were harvested. Total RNA was isolated and subjected to gene expression analysis. A supervised gene cluster analysis revealed a common set of 720 genes that are induced by both Ara-C and ADR but repressed by D9 (Fig 7A and S7 Table). Ingenuity Pathway Analysis (IPA) indicates that the gene categories including “Cellular Movement” and “Cell-To-Cell Signaling and Interaction” appeared among the top five Bio Functions (Fig 7B). In addition, among the top ten canonical pathways which were significantly associated with chemotherapeutic gene response, “Leukocytes Extravasation Signaling”, “IL-8 Signaling” and “Integrin Signaling” were of particular interests as they have been previously linked to AML tumorigenesis (Fig 7C). In particular, the network analysis indicated the involvements of integrins-AKT signalling in chemotherapy induced gene changes (Fig 7D). Our microarray data showed that multiple members of integrins were upregulated by Arac, which were abolished by co-treatment with D9. The same was also observed for multiple ECM ligands of integrins, particularly the laminin family members, and numerous cytokines and their respective receptors, all well known to be associated with cancer progression, self-renewal and drug resistance (Fig 7E, S8–S11 Tables). The microarray results were further validated by quantitative RT-PCR analysis of 15 representative genes of the implicated gene categories (Fig 7F).

Furthermore, in consonance with the above molecular changes, we noticed that Ara-C treatment induced an emergence of a small proportion of TF-1a cells that were attached to the tissue culture plate and exhibited spread and mesenchymal-like morphology, which was reversible upon D9 treatment (Fig 7G). We also found that Ara-C triggered a significant number of migratory, invasive and adherent cells, which were effectively abolished by D9 but not by SAHA. While DAC exerted much weaker effects compared with D9 (Fig 7H, 7I and 7J). These results showed that D9 is able to inhibit the enhanced migration, invasion and adhesion capacities of AML cells induced by chemotherapy. Collectively, these findings demonstrated the potential ability of D9 to target chemoresistance of AML through modulating a wide range of gene sets functionally important in LSC self-renewal, invasion and tumor dissemination.

## Discussion

AML is the most common leukemia of adults, and its incidence increases with age. Although chemotherapy is capable of producing complete remission in almost 70% of patients, many of them will ultimately relapse and succumb to their disease. Thus, there is a great need of new treatment strategies for AML.

In this study, we provide a comprehensive preclinical investigation of D9, an analog of histone methylation inhibitor DZNep in AML. DZNep is a SAM hydrolase inhibitor that inhibits multiple histone methylations and is able to effectively deplete oncogenic PRC2 protein complex and associated H3K27me3. DZNep has been shown to be effective in inducing apoptosis in a variety of cancer cells both in vitro and in vivo and has been used as a useful tool for studying EZH2-mediated gene silencing. In contrast, the specific catalytic inhibitors of EZH2, such as GSK126 or GSK343, though potent in killing some hematological malignant cells such as the diffuse large B-cell lymphoma (DLBCL) that carry EZH2 activating mutations [10, 39], do not seem to be effective in cancer cells overexpressing a wild type EZH2 and only have a modest effect, if any, on EZH2 target gene expression [9, 40].

Like DZNep, D9 inhibits multiple histone methylation marks such as H3K27me3 and H4K20me3 and also depletes EZH2 protein expression in AML cell lines, which was accompanied by effective growth inhibition and apoptosis in both AML cell lines and primary AML cells. It also has a potent anti-tumor effect in vivo with a low level of toxicity. However, the mechanisms governing the pharmacologic response are certainly more complex as the above mentioned changes were seen in both sensitive and resistant AML cell lines. Rather, we believe that the intrinsic differences of AML cells in transcriptional response to the chromatin changes induced by D9 may be responsible for the differential sensitivity to D9. Indeed, we identified gene expression changes that are associated with D9 sensitivity/resistance in AML and these changes indicate the downregulation of a number of key oncogenic pathways and induction of apoptosis, which was validated in both cell lines and primary samples of AML. Thus, the histone methylation inhibitor D9 has produced epigenetic changes that blunted multiple oncogenic signaling pathways in AML simultaneously. Despite the complexity, this actually may provide a much desired advantage in treating cancer cells. Moreover, the molecular changes in p-AKT (S473) and p-ERK (T202/204) as well as the upregulation of Bim and the downregulation of Survivin in response to D9 in an ex vivo test might serve as surrogate markers for evaluating patient’s response to D9 or similar compounds in the future.

A key finding in our study is the observed effect of D9 on leukemia stem cells. This involves the stem-like CD34+CD38- cells in AML which can be induced by chemotherapy. The effect of D9 on chemotherapy-induced CD34+CD38- LSC population correlated well with its ability to deplete the colony forming capacity of LSCs when combined with chemotherapy. It is important to note that in these experiments D9 given at a low nanomolar concentration is sufficient to deplete the LSCs and overcome the chemoresistance, suggesting a potential therapeutic benefit of D9 when combined with chemotherapy in treating AML patients.

Mechanistically, we show that the transcriptional gene expression response in which a large number of genes implicated in cancer stemness, EMT, inflammation, migration and drug resistance are induced by chemotherapy but abolished by D9. In particular, a group of adhesion molecules and their corresponding ligands, multiple cytokines, chemokines, as well as EMT related genes were markedly induced by Ara-C but prevented by D9 co-treatment, which is consistent with the phenotypic changes showing that D9 significantly abrogated the EMT-like conversion as well as increased cell adhesion, migration and invasion following the chemotherapeutic treatment. Numerous studies reported that epithelial-mesenchymal transition [41] cross-talks with metastasis, cancer stem cell and drug resistance [41–44]. Moreover, accumulated evidence suggests that leukemic stem cells (LSCs) in contact with bone marrow (BM) stromal cells and extracellular matrix (ECM) components may acquire resistance to chemotherapy by a process known as cell adhesion-mediated drug resistance (CAM-DR) [45, 46]. Thus, we propose that AML treated with chemotherapeutic agent like Ara-C may utilize the similar mechanism for disease dissemination. The successful mitigation of CAM-DR by D9 suggested the therapeutic potential of D9 to target chemoresistance and AML recurrence.

We also compared D9 with two other FDA approved epigenetic drugs, HDAC inhibitor SAHA and DNA methylation inhibitor DAC in various anti-cancer aspects in AML cell line model. Of significant notice is that our results demonstrated D9 was more potent to suppress AML than SAHA and DAC. Thus, these findings demonstrated a special utility of this class of histone methylation inhibitors for treating AML.

In conclusion, we show that D9 may represent a new generation epigenetic compound that is capable of exerting multifactorial anti-cancer activities including inhibiting multiple oncogenic signaling pathways, targeting LSC and chemoresistance. Regardless of the exact mechanism of D9, its intriguing anti-cancer effects make it a promising cancer drug candidate for further investigation.

## Supporting Information

S1 Table qRT-PCR primers. Table showing the primer sequences from 5’to 3’. (DOCX)

S2 Table EC50 of D9 in solid cancer cell lines. (DOCX)

S3 Table EC50 of D9 in blood cancer cell lines. S2–S3 Tables showing EC50 of D9 measured in 32 solid tumor cell lines and 80 blood cancer cell lines. Data are mean ± SEM; N = 3.
(DOCX)

S4 Table 220 downregulated genes in response to D9 in sensitive cell lines but not in resistant cell lines. (DOCX)

S5 Table 327 upregulated genes in response to D9 in sensitive cell lines but not in resistant cell lines. Three sensitive (MOLM-14, MV4-11 and TF-1) and three resistant cell lines (Mono-Mac-1, KG-1a and THP-1) were treated with D9 at 1 or 5 μM for 48 hours. Total RNA was isolated for microarray and SAM analysis. S4–S5 Tables showing 220 genes were down-regulated and 327 genes were up-regulated upon D9 treatment in sensitive cells relative to resistant cells using 10% false discovery rate (FDR) cut-off.
(DOCX)

S6 Table Information of primary cells from AML patients. Table showing EC50 of D9 in 4 AML patients. Data are mean ± SEM; N = 3.
(DOCX)

S7 Table 720 chemotherapy induced genes. Table showing 720 genesets induced by both Ara-C and ADR which were suppressed by D9.
(DOCX)

S8 Table Normalized microarray data of Integrins. Table showing the averaged values of 46 probes of Integrin members.
(DOCX)

S9 Table Normalized microarray data of Laminins. Table showing the averaged values of 24 probes of Laminins.
(DOCX)

S10 Table Normalized microarray data of cytokines. Table showing the averaged values of 8 probes of cytokines.
(DOCX)

S11 Table Normalized microarray data of the receptors of cytokines. Table showing 8 probes of receptors of cytokines extracted from normalized microarray data of CD34+CD38- double-selected TF-1a cells treated as indicated.
(DOCX)
