# Supplementary material for: Functional Characterization of D9, a Novel Deazaneplanocin A (DZNep) Analog, in Targeting Acute Myeloid Leukemia (AML)
Source: PLoS One. 2015 Apr 30;10(4):e0122983. doi: 10.1371/journal.pone.0122983 (PMC4415792; doi:10.1371/journal.pone.0122983)
Supplement: S1 Table — (DOCX) [file pone.0122983.s001.docx]

**S1 Table. qRT-PCR primers**

| **Gene** | **Primer** | **Sequences (5'-->3')** |
| --- | --- | --- |
| 18S | 18S cybr f | CGAACGTCTGCCCTATCAACTT |
|  | 18S cybr r | ACCCGTGGTCACCATGGTA |
| BCL2L11 | BCL2L11 cybr f | TCAGAACTCATCAGGTACCCACTTATA |
|  | BCL2L11 cybr r | TGGGTCCTAGAAAACAGGTTAGTGA |
| Survivin | Survivin cybr f | GCCAAGAACAAAATTGCAAAGG |
|  | Survivin cybr r | TTTCTCCGCAGTTTCCTCAAA |
| ITGA3 | ITGA3 cybr f | GCCCACCTGGTGTGACTTCT |
|  | ITGA3 cybr r | CGTGGTACTTGGGCATGATCT |
| ITGA5 | ITGA5 cybr f | CAGTGCCGAGTTCACCAAGA |
|  | ITGA5 cybr r | GCCTTGCCAGAAATAGCTTCCT |
| ITGA6 | ITGA6 cybr f | GATCCCGGCCTGTGATTAATATT |
|  | ITGA6 cybr r | CTGGCGGAGGTCAATTCTGT |
| ITGA11 | ITGA11 cybr f | AATATCTCGCAGTCAGCAAACCT |
|  | ITGA11 cybr r | ATGCTACCGTCTGAGTCCTCCTT |
| ITGAM | ITGAM cybr f | GAGTCATTCGCTACGTCATTGG |
|  | ITGAM cybr r | TATTAAGCTCTTGGCGGGATTTC |
| ITGAX | ITGAX cybr f | GCCACCGCCATCCAAAA |
|  | ITGAX cybr r | TCCCTACGGGCCCCATAT |
| ITGB3 | ITGB3 cybr f | GTCCTCCAGCTCATTGTTGATG |
|  | ITGB3 cybr r | GGTCACGCACTTCCAGCTCTA |
| ITGB4 | ITGB4 cybr f | GCAGATCTCCGGTGTACACAAG |
|  | ITGB4 cybr r | GCTTTTTCCCGGCATTGG |
| ITGB5 | ITGB5 cybr f | ACTTCCGGTTGGGATTTGG |
|  | ITGB5 cybr r | GGTGCCGTGTAGGAGAAAGG |
| FN1 | FN1 cybr f | TCGCCATCAGTAGAAGGTAGCA |
|  | FN1 cybr r | TGTTATACTGAACACCAGGTTGCAA |
| LAMA3 | LAMA3 cybr f | AAGCAAGGAGAAATGCAATGATG |
|  | LAMA3 cybr r | CAACCAAGCGCCCCTTTT |
| LAMB3 | LAMB3 cybr f | CCTTATGGATTTAGTGTCTGGGATTC |
|  | LAMB3 cybr r | TTGTGATCTCCCCCACCTCTT |
| COL2A1 | COL2A1 cybr f | GTACTTTCCAATCTCAGTCACTCTAGGA |
|  | COL2A1 cybr r | GGTGGGATGAATGGACATCAG |
| COL7A1 | COL7A1 cybr f | CCTGTCACCCTTTTGTCTATGGT |
|  | COL7A1 cybr r | CTCACGGGTCCCAAAACG |
| COL9A2 | COL9A2 cybr f | GTGGTGTCGATGGAGTCGAA |
|  | COL9A2 cybr r | CGAATGATGCTCGCTGGAA |
| COL24A1 | COL24A1 cybr f | GGCATATACATCTTAGCCGGTGAT |
|  | COL24A1 cybr r | AAAGAGCACCAAGCAACACAAA |
| ECM1 | ECM1 cybr f | GCCACACAAACCGCCTAGA |
|  | ECM1 cybr r | AGAATCGGCTCATTGCTTCCT |
| ICAM1 | ICAM1 cybr f | TGGCCCTCCATAGACATGTGT |
|  | ICAM1 cybr r | TGGCATCCGTCAGGAAGTG |
| PECAM1 | PECAM1 cybr f | CAGGCGCCGGGAGAA |
|  | PECAM1 cybr r | CACAAGTCACCGTTGAGAAACC |
| VCAN | VCAN cybr f | GAATGGCCAGTGGAATGATGTT |
|  | VCAN cybr r | CCGCAAGCGACTGTTCCTT |
| CDH1 | CDH1 cybr f | ACAGCCCCGCCTTATGATT |
|  | CDH1 cybr r | TCGGAACCGCTTCCTTCA |
| Lin28 | Lin28 cybr f | TCCTCATACCCACTTTTGGGATA |
|  | Lin28 cybr r | GCCATCATCATTACCCATTGC |
| CD44 | CD44 cybr f | CATAGAAGGGCACGTGGTGAT |
|  | CD44 cybr r | TTTGCTGCACAGATGGAGTTG |
| MMP2 | MMP2 cybr f | TGAGCTATGGACCTTGGGAGAA |
|  | MMP2 cybr r | CCATCGGCGTTCCCATAC |
| MMP9 | MMP9 cybr f | GGACGATGCCTGCAACGT |
|  | MMP9 cybr r | CAAATACAGCTGGTTCCCAATCT |
| TGFBI | TGFBI cybr f | AGCCATTGACCTTTTCAGACAAG |
|  | TGFBI cybr r | GGTCAACCGCTCACTTCCA |
| TGFBR2 | TGFBR2 cybr f | CAACCACCAGGGCATCCA |
|  | TGFBR2 cybr r | TCGTGGTCCCAGCACTCA |
| IL-8 | IL-8 cybr f | CACCGGAAGGAACCATCTCA |
|  | IL-8 cybr r | AGAGCCACGGCCAGCTT |
| CCL5 | CCL5 cybr f | GGTCAAGGATGCCAAAGAGAGA |
|  | CCL5 cybr r | GCCGGGAGTCATACAGGAAA |
| CCL23 | CCL23 cybr f | TGCTCCAAGCCAGGTGTCA |
|  | CCL23 cybr r | CACTGGGTTTGGCACAGACTT |
