# Supplementary material for: Functional Characterization of D9, a Novel Deazaneplanocin A (DZNep) Analog, in Targeting Acute Myeloid Leukemia (AML)
Source: PLoS One. 2015 Apr 30;10(4):e0122983. doi: 10.1371/journal.pone.0122983 (PMC4415792; doi:10.1371/journal.pone.0122983)
Supplement: S2 Table — (DOCX) [file pone.0122983.s002.docx]

**S2 Table. EC50 of D9 in solid cancer cell lines**

| **Cell line** | **EC50 (M)** |
| --- | --- |
| HCT-15 | 5.46E-07 |
| HCT116 | 1.09E-06 |
| LOXIMVI | 1.52E-06 |
| U251 | 1.54E-06 |
| UO31 | 2.72E-06 |
| IGR-OV1 | 7.88E-06 |
| SW620 | 8.18E-06 |
| KM12 | 8.46E-06 |
| COLO 205 | 9.51E-06 |
| OVCAR5 | 1.23E-05 |
| OVCAR3 | 1.39E-05 |
| Sk-Mel-2 | 1.53E-05 |
| HT-29 | 1.77E-05 |
| OVCAR8 | 1.84E-05 |
| SN12C | 1.98E-05 |
| MCF 7 | 2.45E-05 |
| T47D | 2.56E-05 |
| EKVX | 2.80E-05 |
| A549 | 3.50E-05 |
| Du145 | 3.69E-05 |
| HCC 2998 | 3.81E-05 |
| PC3 | 4.00E-05 |
| ACHN | 4.31E-05 |
| NCI-ADR-RES | 4.42E-05 |
| Caki-1 | 5.74E-05 |
| NCI-H23 | 9.58E-05 |
| MDA-MB-231 | 1.19E-04 |
| SF539 | 3.01E-04 |
| NCI-H226 | 6.61E-04 |
| RXF393 | 9.73E-04 |
| UACC257 | 1.72E-03 |
| BT-549 | 3.85E-03 |
