# Supplementary material for: Functional Characterization of D9, a Novel Deazaneplanocin A (DZNep) Analog, in Targeting Acute Myeloid Leukemia (AML)
Source: PLoS One. 2015 Apr 30;10(4):e0122983. doi: 10.1371/journal.pone.0122983 (PMC4415792; doi:10.1371/journal.pone.0122983)
Supplement: S3 Table — S2–S3 Tables showing EC50 of D9 measured in 32 solid tumor cell lines and 80 blood cancer cell lines. Data are mean ± SEM; N = 3. (DOCX) [file pone.0122983.s003.docx]

**S3 Table. EC50 of D9 in blood cancer cell lines**

| **Cell line** | **EC50 (M)** |
| --- | --- |
| MV-4-11 | 1.07E-07 |
| CEM-CM3 | 1.32E-07 |
| HL-60 | 1.51E-07 |
| I2.1 | 1.64E-07 |
| EOL-1 | 1.76E-07 |
| Sup-B15 | 2.50E-07 |
| DOHH2 | 3.53E-07 |
| MHH-CALL2 | 4.27E-07 |
| BC-3 | 4.98E-07 |
| NK-92-MI | 5.20E-07 |
| RS4(11) | 5.20E-07 |
| GA10(Clone4) | 5.26E-07 |
| 8E5 | 5.28E-07 |
| RL | 6.22E-07 |
| TOLEDO | 6.22E-07 |
| NK92 | 6.36E-07 |
| Farage | 6.81E-07 |
| U937 | 7.82E-07 |
| GDM | 8.10E-07 |
| CCRF-HSB | 8.12E-07 |
| MC116 | 8.84E-07 |
| CESS | 1.01E-06 |
| TALL-104 | 1.14E-06 |
| P116.CL39 | 1.37E-06 |
| ML-2 | 1.42E-06 |
| MOLT-4 | 1.47E-06 |
| MEG-01 | 1.71E-06 |
| MO | 1.75E-06 |
| HUT78 | 1.91E-06 |
| EB3 | 2.06E-06 |
| SR | 2.30E-06 |
| CA46 | 2.32E-06 |
| J45.01 | 2.36E-06 |
| GA-10 | 2.54E-06 |
| KE-37 | 2.57E-06 |
| Loucy | 2.63E-06 |
| EB2 | 2.66E-06 |
| KG-1 | 2.75E-06 |
| MJ | 3.14E-06 |
| KASUMI-4 | 3.15E-06 |
| EB1 | 3.82E-06 |
| MOLT-3 | 3.98E-06 |
| D1.1 | 4.15E-06 |
| P3HR-1 | 4.33E-06 |
| BC-1 | 4.67E-06 |
| HT | 4.75E-06 |
| Jiyoye | 4.75E-06 |
| BCP-1 | 5.14E-06 |
| Kasumi-1 | 6.15E-06 |
| SUP-T1 | 6.37E-06 |
| KG1a | 6.37E-06 |
| J.1WT | 6.39E-06 |
| DG75 | 6.66E-06 |
| NCI-H526 | 6.75E-06 |
| NC-37 | 6.80E-06 |
| MSLT-4 | 6.89E-06 |
| CCRF-CEM | 7.34E-06 |
| CEM/C1 | 7.39E-06 |
| ST486 | 7.57E-06 |
| K-562 | 8.68E-06 |
| P116 | 9.13E-06 |
| JRT3.T35 | 9.13E-06 |
| GK-5 | 1.02E-05 |
| ARH-77 | 1.05E-05 |
| I9.2 | 1.07E-05 |
| Ramos | 1.09E-05 |
| Daudi | 1.25E-05 |
| REH | 1.34E-05 |
| BDCM | 1.79E-05 |
| RAMOS.2Gb.4c10 | 2.03E-05 |
| H9 | 2.04E-05 |
| NCI-H82 | 2.48E-05 |
| K562 | 2.87E-05 |
| Raji | 3.28E-05 |
| CEM/C2 | 3.51E-05 |
| 2B8 | 3.53E-05 |
| RPMI-8226 | 5.54E-05 |
| Colo-704 | 6.30E-05 |
| CCRF-CEM | 6.73E-05 |
| SNU-16 | 1.83E-04 |
