# Supplementary material for: Functional Characterization of D9, a Novel Deazaneplanocin A (DZNep) Analog, in Targeting Acute Myeloid Leukemia (AML)
Source: PLoS One. 2015 Apr 30;10(4):e0122983. doi: 10.1371/journal.pone.0122983 (PMC4415792; doi:10.1371/journal.pone.0122983)
Supplement: S4 Table — (DOCX) [file pone.0122983.s004.docx]

**S4 Table. 220 downregulated genes in response to D9 treatment in sensitive cell lines but not in resistant cell lines**

| IGLL1 |
| --- |
| P2RY2 |
| LAMC2 |
| LOC285501 |
| FIGLA |
| COL15A1 |
| AVPR1B |
| LOC652526 |
| CD34 |
| CD34 |
| FAM125B |
| PRICKLE1 |
| LST1 |
| RALGPS2 |
| LOC643216 |
| FOSB |
| LOC653310 |
| ALDH7A1 |
| JMJD2A |
| LOC100130809 |
| PLGLB1 |
| MIR224 |
| APOD |
| FLJ46309 |
| KLHL35 |
| ASCC3 |
| LOC731039 |
| SPINK9 |
| LOC645167 |
| PIGN |
| IGLL3 |
| LOC642563 |
| CLEC12A |
| FXYD4 |
| LTB |
| HS.583323 |
| CPLX1 |
| AFARP1 |
| MIR1290 |
| SPZ1 |
| STATH |
| MT1M |
| LST1 |
| LST1 |
| RGPD1 |
| AXL |
| CHRDL1 |
| ADAM32 |
| ZNF568 |
| IL26 |
| SOX8 |
| CEP97 |
| CBWD7 |
| NACC2 |
| LOC100128010 |
| HS.555115 |
| SNIP |
| LOC643453 |
| CACNB2 |
| HIST1H4A |
| HYDIN |
| ERCC-00031 |
| VWA5B1 |
| CALCB |
| LOC648868 |
| BCAS4 |
| ATP5I |
| LOC283663 |
| CR1 |
| LOC441193 |
| CHMP6 |
| LOC730417 |
| LOC440361 |
| TNF |
| LOC729852 |
| HIST1H2BH |
| SETD7 |
| C15ORF58 |
| COLEC11 |
| LOC388707 |
| GPR98 |
| GSDMC |
| GLP2R |
| GNL3 |
| IL28B |
| LOC401074 |
| ZNF541 |
| C14ORF37 |
| ST6GAL2 |
| ID1 |
| LIMS2 |
| PTH |
| SNORA60 |
| LOC729249 |
| GAP43 |
| LOC401010 |
| LOC653980 |
| IKBKAP |
| CSF3R |
| LOC728533 |
| SPRR2B |
| BLK |
| AP1G2 |
| HS.562219 |
| OR51F1 |
| LOC728804 |
| LOC100130241 |
| LOC730351 |
| CEP63 |
| HPDL |
| SH2D5 |
| PLA2G3 |
| PNMA6A |
| OR4M2 |
| LOC648415 |
| C17ORF75 |
| LOC100130516 |
| OR7D2 |
| WNT11 |
| DOCK7 |
| CLDN15 |
| SSX7 |
| AMY1A |
| EBF3 |
| ANKRD18A |
| C1ORF102 |
| LOC338829 |
| MDK |
| GLTPD1 |
| KPRP |
| C21ORF41 |
| LOC654032 |
| HOXA10 |
| ALG11 |
| OR5M8 |
| FIT1 |
| ALDH1B1 |
| LOC642965 |
| EPO |
| DNAJB12 |
| NR3C2 |
| LOC645263 |
| LOC100130982 |
| LOC400950 |
| LOC649201 |
| LOC653555 |
| ST8SIA1 |
| C16ORF65 |
| LOC652286 |
| KTELC1 |
| ANKHD1-EIF4EBP3 |
| TSSK4 |
| FAM184A |
| LOC100132859 |
| SLC25A34 |
| ZNF232 |
| PXMP3 |
| MFNG |
| PTPDC1 |
| FAM134C |
| FLJ44290 |
| MMP25 |
| LOC650657 |
| FAM163B |
| ZNF783 |
| XKRY |
| BMP2 |
| CUEDC1 |
| HS.255966 |
| HS.473255 |
| BCORL2 |
| CD86 |
| LOC653468 |
| LOC100130825 |
| SEMA4G |
| INHBE |
| CDRT15L2 |
| LOC648398 |
| LOC100131660 |
| CCNA1 |
| LOC642477 |
| KCNJ14 |
| RASSF10 |
| LOC645001 |
| LOC642426 |
| TIMM17A |
| GHSR |
| HIST1H3D |
| SERPINA7 |
| LOC729595 |
| JMJD4 |
| OR2T5 |
| RPL29 |
| FGF14 |
| LOC643468 |
| PPIL3 |
| HYPB |
| PNMA3 |
| ZNF100 |
| GABRE |
| HS.582338 |
| LOC440345 |
| GATA2 |
| PLXNA1 |
| SPTBN1 |
| HS.553187 |
| LOC730121 |
| CCDC85A |
| NUDT19 |
| THBD |
| SLC18A2 |
| SUPT3H |
| PPAN-P2RY11 |
| EMR4 |
| HS.25555 |
| GNA12 |
| OR5T2 |
| NAALADL2 |
| IL9 |
| EPS8L2 |
