# Supplementary material for: Functional Characterization of D9, a Novel Deazaneplanocin A (DZNep) Analog, in Targeting Acute Myeloid Leukemia (AML)
Source: PLoS One. 2015 Apr 30;10(4):e0122983. doi: 10.1371/journal.pone.0122983 (PMC4415792; doi:10.1371/journal.pone.0122983)
Supplement: S5 Table — Three sensitive (MOLM-14, MV4-11 and TF-1) and three resistant cell lines (Mono-Mac-1, KG-1a and THP-1) were treated with D9 at 1 or 5 μM for 48 hours. Total RNA was isolated for microarray and SAM analysis. S4–S5 Tables showing 220 genes were down-regulated and 327 genes were up-regulated upon D9 treatment in sensitive cells relative to resistant cells using 10% false discovery rate (FDR) cut-off. (DOCX) [file pone.0122983.s005.docx]

**S5 Table. 327 upregulated genes in response to D9 treatment in sensitive cell lines but not in resistant cell lines**

| SPHK1 |
| --- |
| LOC653715 |
| KIAA1245 |
| TRIM13 |
| SOCS4 |
| NPVF |
| RPP30 |
| OAT |
| CACNA1F |
| DUOX2 |
| MIR510 |
| MUT |
| H19 |
| MAMLD1 |
| LOC100133714 |
| SATB1 |
| LOC728116 |
| PTPLB |
| LOC399959 |
| HS.542001 |
| KEAP1 |
| SMN1 |
| LOC647013 |
| LOC652676 |
| TKTL1 |
| LOC440015 |
| LOC650120 |
| CD47 |
| SLIT3 |
| KIAA0907 |
| ANKRD34B |
| LOC642423 |
| EVX2 |
| AIRE |
| RTP4 |
| LOC645217 |
| LOC389607 |
| NDRG2 |
| EDEM1 |
| KIAA1333 |
| C6ORF205 |
| HS.536620 |
| ZP1 |
| MYH6 |
| SERP2 |
| PPP1R1C |
| LOC391160 |
| NAP1L1 |
| GJB3 |
| TOR1AIP1 |
| LOC100134648 |
| LOC731718 |
| TIFAB |
| SRGAP3 |
| AADACL2 |
| LOC100133949 |
| COPB1 |
| MIR548I4 |
| HLA-DOB |
| SP110 |
| CD40 |
| CHKA |
| MYH14 |
| HS.541921 |
| HSPA12A |
| BRF1 |
| SCARNA2 |
| USP21 |
| OR7A5 |
| LOC100132588 |
| PCDHA4 |
| KIAA1598 |
| BUB3 |
| SP3 |
| CA1 |
| CCNE2 |
| HS.538525 |
| HS.568690 |
| VEGFB |
| DEPDC5 |
| HS.546177 |
| SPIN4 |
| FAM153C |
| C4ORF32 |
| KIAA1239 |
| LOC644508 |
| SORCS2 |
| CAPZA1 |
| FAM135A |
| PRAMEF10 |
| LCE1E |
| KRTHA5 |
| IDS |
| CALML4 |
| RPS8 |
| METTL1 |
| CSAG2 |
| SNAP25 |
| CAMK4 |
| LOC100134370 |
| HS.559860 |
| OFCC1 |
| LOC730417 |
| CYP2W1 |
| FLJ20397 |
| LOC644188 |
| MAN1A1 |
| LOC644204 |
| ADAM19 |
| TREX2 |
| MOGAT2 |
| RAB40AL |
| NPTX2 |
| ALDOB |
| CYP26B1 |
| MIR15B |
| GJB2 |
| HS.525922 |
| FLJ14213 |
| YPEL3 |
| FCN2 |
| SIGLEC10 |
| TRIM71 |
| CLEC7A |
| LOC100130264 |
| MS4A7 |
| LOC401351 |
| SYNE1 |
| RNF144A |
| HS.572735 |
| LOC100133629 |
| CCDC67 |
| C9ORF53 |
| CCR6 |
| ID2 |
| BOLA3 |
| SEC24A |
| RNASE4 |
| BACE1 |
| HAUS5 |
| LOC100130648 |
| HS.571245 |
| CMPK1 |
| ZNF345 |
| OCLN |
| LOC100134292 |
| MAGEA8 |
| NUDT21 |
| LOC647881 |
| MINPP1 |
| LPIN1 |
| EFCAB6 |
| GUCY2G |
| HOXA9 |
| SDHALP1 |
| LOC100129365 |
| ROPN1 |
| LOC730101 |
| LOC100133005 |
| RAP2C |
| PLGLA1 |
| LOC286177 |
| ARHGAP28 |
| ATP13A5 |
| MRGPRF |
| LOC648841 |
| KIAA0367 |
| AKAP11 |
| CLIC6 |
| TNRC6B |
| PLAGL1 |
| CXCR5 |
| SLC7A14 |
| HS.294103 |
| TNRC18 |
| PHTF2 |
| ISG20 |
| RASA1 |
| CCNG2 |
| HS.224449 |
| UGCGL1 |
| NBR1 |
| COMP |
| HS.542934 |
| SGPP2 |
| LOC650990 |
| PCDHB7 |
| LOC653086 |
| LOC100129426 |
| DKFZP434N035 |
| LOC728734 |
| LOC728465 |
| NRF1 |
| LOC440145 |
| DNAJB4 |
| RBM46 |
| LOC642902 |
| PION |
| LOC100130883 |
| 4-Sep |
| LOC441344 |
| C20ORF173 |
| AOAH |
| ABCB1 |
| CASP8AP2 |
| LOC642628 |
| MIR181B2 |
| MGC22014 |
| MIR1914 |
| CCDC83 |
| MS4A7 |
| LOC124220 |
| LOC387882 |
| CREB5 |
| DMTF1 |
| WWOX |
| LOC652674 |
| SPINK5L3 |
| CSAG3 |
| RNASEL |
| LRRC70 |
| MIR148A |
| CASZ1 |
| SHC2 |
| IGFBP6 |
| LOC100129882 |
| MAPK10 |
| MDM1 |
| C12ORF76 |
| CGB1 |
| GRIP2 |
| IDS |
| HS.137216 |
| CSRNP2 |
| APOL3 |
| PEBP4 |
| CREB5 |
| LRRC3B |
| TMEM92 |
| MIR1267 |
| INPP5F |
| TMX3 |
| AKT3 |
| MID1 |
| LOC644952 |
| PSMB9 |
| LOC649700 |
| MX1 |
| L3MBTL3 |
| LOC730322 |
| ERCC-00061 |
| TSC22D2 |
| SAMSN1 |
| HSD11B1 |
| HS.565314 |
| SELL |
| CD302 |
| FRAS1 |
| PMAIP1 |
| FAM57B |
| ZNF713 |
| PGCP |
| CAPN5 |
| YPEL1 |
| MST4 |
| TJP2 |
| CAMK1D |
| EFNB3 |
| EMR3 |
| DLEU2L |
| LOC648905 |
| BRAP |
| HS.232517 |
| NRARP |
| CLDND1 |
| RASL11B |
| KIAA0692 |
| BACH1 |
| PGCP |
| C1ORF55 |
| LOC646041 |
| MAGEB4 |
| CRABP1 |
| TUFT1 |
| IFNA5 |
| EYA4 |
| HS.563340 |
| SP4 |
| ARFGAP3 |
| ADCYAP1 |
| MLL5 |
| LOC649238 |
| LOC650405 |
| CD209 |
| RHOBTB1 |
| LOC649366 |
| DR1 |
| IL4I1 |
| OR7D4 |
| HS.216701 |
| ZNF658B |
| DNAJB4 |
| LOC730376 |
| CD5 |
| MATN2 |
| LARP6 |
| FLJ37786 |
| LOC100132496 |
| FAM70A |
| KIAA1618 |
| MCHR2 |
| CLEC10A |
| FBXL3 |
| SLCO1A2 |
| LOC645378 |
| FAM76B |
| LOC152663 |
| FLJ43950 |
| LOC441070 |
| LOC441052 |
| OR1L6 |
| IFIT3 |
| SLC44A3 |
| KCNC3 |
| TEX15 |
| HS.541066 |
| LOC120376 |
