# Supplementary material for: Functional Characterization of D9, a Novel Deazaneplanocin A (DZNep) Analog, in Targeting Acute Myeloid Leukemia (AML)
Source: PLoS One. 2015 Apr 30;10(4):e0122983. doi: 10.1371/journal.pone.0122983 (PMC4415792; doi:10.1371/journal.pone.0122983)
Supplement: S6 Table — Table showing EC50 of D9 in 4 AML patients. Data are mean ± SEM; N = 3. (DOCX) [file pone.0122983.s006.docx]

**S6 Table. Information of primary cells from AML patients**

| **Patient ID** | **EC50 of D9 (µM)** | **FAB subtypes** | **Sources** |
| --- | --- | --- | --- |
| AD267 | 4.32 | M1 | Bone Marrow (BM) |
| AD353 | 0.96 | MDS | Bone Marrow (BM) |
| AD362a | 4.06 | MDS | Peripheral Blood (PB) |
| AD409 | 13.43 | M5 | Bone Marrow (BM) |
