# Supplementary material for: Functional Characterization of D9, a Novel Deazaneplanocin A (DZNep) Analog, in Targeting Acute Myeloid Leukemia (AML)
Source: PLoS One. 2015 Apr 30;10(4):e0122983. doi: 10.1371/journal.pone.0122983 (PMC4415792; doi:10.1371/journal.pone.0122983)
Supplement: S7 Table — Table showing 720 genesets induced by both Ara-C and ADR which were suppressed by D9. (DOCX) [file pone.0122983.s007.docx]

**S7 Table. 720 chemotherapy induced genes**

| **Symbol** | **DMSO** | **D-9** | **Ara-C** | **D9+Ara-C** | **DMSO** | **D9** | **ADR** | **D9+ADR** |
| --- | --- | --- | --- | --- | --- | --- | --- | --- |
| UGT2B7 | -1.25 | 0.45 | 1.49 | 0.12 | -0.04 | -0.36 | 1.42 | -0.15 |
| BIN1 | 0.01 | -0.24 | 0.73 | 0.54 | 0.13 | -0.08 | 1.08 | 0.88 |
| FCGR2A | -0.10 | -0.30 | 1.15 | 0.20 | 0.48 | -0.06 | 0.95 | 0.37 |
| ADAMTSL4 | -0.63 | -1.29 | 0.86 | 0.13 | -0.32 | -0.50 | 1.30 | 0.84 |
| QPCT | -0.39 | 0.01 | 1.39 | 1.07 | -0.14 | -0.02 | 1.11 | 0.41 |
| PECAM1 | 0.11 | -0.15 | 0.89 | 0.31 | 0.32 | 0.30 | 0.80 | -0.03 |
| GABARAPL1 | 0.30 | -0.87 | 0.44 | -0.27 | 0.30 | -0.48 | 0.51 | 0.03 |
| TMEM55A | 0.04 | -0.41 | 1.08 | -0.04 | 0.67 | 0.98 | 1.18 | 0.95 |
| CASP8 | 0.35 | -0.24 | 0.58 | -0.01 | 0.75 | 0.20 | 1.07 | 0.55 |
| ACSM3 | -0.13 | -0.91 | 0.67 | 0.53 | -0.09 | -0.70 | 0.88 | 0.09 |
| ACSM3 | -0.58 | -0.84 | 0.71 | 0.30 | 0.00 | -0.57 | 1.04 | 0.23 |
| DACT3 | -0.06 | -0.91 | 1.32 | 0.52 | -0.01 | -0.85 | 1.33 | 0.79 |
| MICAL1 | -0.06 | -0.38 | 1.12 | 0.54 | 0.03 | -0.03 | 1.50 | 1.02 |
| ALOX5AP | -1.14 | 0.09 | 2.24 | 1.09 | -0.09 | 0.10 | 2.00 | 1.20 |
| EPSTI1 | -0.52 | -1.85 | 1.37 | 0.21 | -0.20 | -1.40 | 1.24 | 0.02 |
| CD69 | 0.21 | -1.30 | 1.09 | -0.15 | 0.19 | -1.28 | 0.42 | -0.98 |
| C20orf197 | -0.52 | -0.64 | 2.84 | 1.68 | 1.80 | -0.01 | 2.70 | 0.13 |
| LOC606724 | -0.26 | -1.07 | 0.97 | 0.09 | 0.60 | -0.09 | 1.04 | 0.24 |
| UGP2 | -0.68 | -0.33 | 0.30 | 0.20 | -0.14 | -0.88 | 0.76 | 0.14 |
| LOC100128975 | -0.40 | -0.51 | 0.46 | -0.03 | 0.16 | -0.47 | 0.83 | 0.49 |
| FCER1G | 0.01 | -0.44 | 1.21 | 0.42 | 0.47 | -0.07 | 0.96 | 0.22 |
| SLC26A6 | -0.66 | -0.12 | 0.33 | -0.57 | -1.18 | -0.21 | 0.90 | -0.09 |
| SLFN11 | 0.04 | -0.67 | 0.84 | 0.53 | -0.97 | -1.45 | -0.26 | -0.53 |
| BBS7 | 0.02 | -0.55 | 0.39 | 0.31 | 0.45 | -0.68 | 0.61 | 0.37 |
| NXT2 | 0.35 | -0.01 | 0.93 | 0.29 | -0.93 | -1.00 | -0.43 | -0.75 |
| MS4A6A | 0.22 | -1.45 | 2.54 | 1.13 | 0.69 | -0.21 | 1.65 | 0.55 |
| KIAA1949 | 0.05 | -0.19 | 0.56 | 0.01 | 0.27 | -0.15 | 0.64 | 0.18 |
| CAMTA1 | 0.09 | -0.59 | 0.46 | -0.34 | 0.02 | -0.61 | 0.19 | -0.92 |
| LOC100128252 | 0.21 | -0.12 | 1.14 | -0.28 | 0.81 | -0.42 | 1.25 | 0.28 |
| ZMAT3 | 0.09 | -0.52 | 1.28 | 0.29 | 0.29 | -0.43 | 0.66 | 0.00 |
| LY96 | -0.15 | -2.98 | 0.45 | -0.03 | 0.23 | -3.02 | 1.00 | 0.03 |
| VAMP5 | 0.02 | -0.45 | 1.24 | 0.73 | -0.03 | -0.27 | 1.05 | 0.31 |
| SMAD7 | -0.30 | -0.31 | 0.59 | 0.41 | 0.21 | 0.70 | 0.73 | 0.06 |
| ZNF564 | -0.06 | -0.57 | 0.49 | 0.35 | 0.04 | -0.76 | 0.34 | -0.21 |
| EAF2 | -0.14 | -0.24 | 0.62 | 0.40 | -0.11 | 0.12 | 0.60 | 0.04 |
| PVRL2 | 0.07 | -0.63 | 0.83 | -0.07 | 0.37 | -0.64 | 0.66 | -0.33 |
| CD44 | 0.13 | -0.33 | 1.24 | 0.59 | 0.11 | -0.03 | 1.28 | 0.40 |
| EFNB2 | 0.22 | -1.43 | 0.77 | 0.02 | 0.29 | 0.12 | 0.97 | 0.26 |
| PWWP2B | -0.38 | -0.07 | 0.67 | 0.45 | -0.20 | -0.08 | 0.23 | 0.16 |
| SNORA4 | 0.13 | -1.21 | 0.42 | -0.04 | 0.19 | -0.24 | 0.47 | -0.38 |
| NMU | -0.80 | -0.88 | 0.71 | 0.10 | -0.89 | -0.85 | 0.97 | 0.44 |
| CBLB | -0.17 | -0.90 | 0.56 | 0.00 | 0.27 | -0.08 | 1.26 | 0.57 |
| ACVRL1 | -0.82 | -0.43 | 0.67 | 0.60 | -0.88 | 0.01 | 1.11 | 1.02 |
| ITGB7 | -0.68 | -0.57 | 0.97 | 0.65 | -0.54 | -0.55 | 1.11 | 0.59 |
| LOC400304 | 0.10 | -0.41 | 0.78 | 0.40 | -2.03 | -2.39 | -0.79 | -1.39 |
| DOK2 | -0.02 | -0.33 | 1.19 | 0.74 | -0.52 | -0.50 | 0.57 | 0.40 |
| SMARCA1 | 0.24 | -0.86 | 0.54 | -0.02 | -0.26 | -0.93 | 0.49 | -0.01 |
| C10orf33 | -0.39 | -0.32 | 1.10 | 0.51 | 0.04 | 0.02 | 0.61 | 0.04 |
| LGALS1 | 0.09 | -0.84 | 0.64 | -0.19 | 0.56 | -0.18 | 1.23 | 0.22 |
| IRF1 | -0.66 | -1.13 | 0.43 | -0.08 | 0.13 | -0.14 | 0.92 | 0.73 |
| SLCO2B1 | 0.12 | -1.06 | 1.08 | -0.02 | 0.02 | -1.00 | 0.46 | -0.98 |
| SLCO2B1 | 0.03 | -1.09 | 0.70 | -0.22 | 0.20 | -1.03 | 0.50 | -0.46 |
| PYCARD | -0.08 | -0.57 | 0.85 | 0.56 | 0.07 | -0.38 | 0.75 | 0.48 |
| ADAM15 | -0.36 | -0.44 | 0.56 | 0.12 | 0.01 | -0.12 | 0.60 | 0.46 |
| RAB37 | -0.44 | -0.57 | 1.68 | 0.98 | -2.29 | -0.19 | 1.42 | 0.58 |
| HAVCR2 | 0.04 | -0.63 | 2.77 | 0.97 | 0.08 | -0.03 | 1.15 | 0.42 |
| TRAPPC9 | -0.10 | -0.18 | 0.64 | 0.06 | 0.50 | -0.11 | 0.94 | 0.31 |
| ABHD2 | -0.29 | -0.85 | 0.52 | 0.46 | 0.17 | -0.09 | 0.87 | 0.82 |
| ADAM11 | -0.51 | -1.37 | 1.12 | 0.36 | -0.36 | -0.28 | 1.37 | 0.91 |
| RP5-1022P6.2 | -0.03 | -0.08 | 0.65 | -0.03 | -0.39 | -0.34 | 0.76 | 0.31 |
| TREML2 | -0.38 | -1.32 | 1.60 | 0.66 | 0.48 | -0.19 | 1.36 | 0.89 |
| GPR65 | 0.16 | 0.12 | 1.74 | -0.06 | -0.82 | -0.98 | 0.72 | 0.01 |
| LY6G5C | -0.02 | -1.36 | 0.58 | -0.17 | 0.00 | -1.14 | 1.24 | 0.67 |
| S100A10 | -0.01 | -0.50 | 0.95 | 0.42 | -0.13 | -0.33 | 0.68 | 0.04 |
| PTPN6 | -0.06 | -0.51 | 0.65 | 0.18 | 0.17 | 0.19 | 0.77 | 0.40 |
| LST1 | 0.12 | -1.75 | 2.38 | 1.01 | -0.12 | 0.33 | 1.65 | 0.74 |
| SGK3 | 0.16 | -0.37 | 0.39 | -0.26 | 0.03 | -0.66 | 0.24 | -0.20 |
| SERPING1 | -0.01 | -1.52 | 1.47 | 0.89 | -0.07 | -0.62 | 1.08 | 0.21 |
| FGD3 | -0.22 | -0.38 | 0.67 | 0.06 | -0.07 | -0.32 | 0.58 | 0.49 |
| SGIP1 | -0.89 | -1.01 | 1.18 | 0.75 | -0.98 | -0.50 | 1.62 | 1.40 |
| TSPAN9 | 0.26 | -0.67 | 0.80 | -0.10 | 0.54 | -0.42 | 0.93 | 0.30 |
| PM20D2 | 0.93 | 0.02 | 0.95 | -0.02 | -0.48 | -1.16 | -0.18 | -1.32 |
| SDCBP | -0.07 | -0.28 | 0.53 | 0.24 | 0.22 | -0.12 | 0.98 | 0.42 |
| LOC284422 | 0.29 | -0.60 | 1.30 | 0.08 | -0.03 | -0.64 | 1.81 | 0.76 |
| OPRL1 | -0.29 | -0.69 | 0.55 | 0.21 | 0.07 | -0.07 | 0.98 | 0.68 |
| HAPLN4 | 0.00 | -1.56 | 1.08 | 0.55 | 0.00 | -0.35 | 0.84 | 0.67 |
| CD52 | -0.63 | -0.54 | 1.50 | 0.78 | -0.20 | 0.03 | 0.98 | 0.54 |
| SLC22A17 | -0.20 | -0.23 | 0.97 | 0.85 | -0.46 | -0.43 | 0.52 | 0.19 |
| F2RL3 | -0.49 | -1.12 | 1.23 | 0.62 | 0.58 | -0.72 | 0.79 | 0.25 |
| ECM1 | -0.88 | -0.60 | 1.64 | 0.60 | -0.24 | -1.82 | 0.71 | -0.04 |
| CNR1 | -1.16 | -1.32 | 1.01 | 0.78 | -0.70 | -0.33 | 0.64 | 0.37 |
| SYNJ2 | -0.21 | -1.71 | 1.49 | 0.49 | 0.02 | -0.87 | 1.22 | 0.99 |
| EPHA1 | -0.45 | -0.39 | 1.15 | 0.63 | -1.28 | -0.02 | 0.91 | 0.35 |
| MCTP1 | 0.23 | -0.73 | 0.71 | -0.05 | 0.58 | 0.16 | 1.00 | 0.05 |
| BASP1 | -0.18 | -0.22 | 0.79 | 0.16 | 0.72 | 0.01 | 1.27 | -0.11 |
| ZNF439 | -0.35 | -0.97 | 1.28 | -0.02 | -0.35 | -1.18 | 1.18 | 0.44 |
| BMPER | 0.54 | -0.92 | 1.51 | 0.61 | 0.77 | -0.78 | 1.23 | 0.68 |
| RAB3C | -0.01 | -0.89 | 0.72 | 0.01 | 0.25 | -0.33 | 0.88 | 0.27 |
| NCF4 | -0.33 | -0.51 | 1.06 | 0.35 | -0.08 | -0.25 | 0.95 | 0.75 |
| CST1 | 0.18 | -2.03 | 2.48 | 0.94 | 0.99 | -1.11 | 1.51 | 0.70 |
| ZNF812 | -0.92 | -0.55 | 0.31 | -0.01 | 0.04 | -0.65 | 0.51 | 0.38 |
| LOC100129905 | 0.00 | -0.64 | 0.45 | -0.08 | 0.90 | -0.34 | 1.05 | 0.48 |
| FAM13B | -0.18 | -0.21 | 0.61 | -0.16 | 0.04 | -0.01 | 0.87 | 0.44 |
| GCC2 | -0.69 | 0.06 | 0.34 | -0.28 | -0.31 | 0.02 | 0.94 | -0.30 |
| FAM105A | -0.16 | 0.02 | 1.05 | 0.84 | 0.39 | -0.02 | 0.61 | 0.57 |
| ITGB2 | -0.40 | -0.53 | 0.29 | 0.10 | 0.06 | -0.10 | 0.66 | 0.49 |
| RASL10A | -0.36 | -0.40 | 1.08 | 0.72 | 0.03 | 0.08 | 1.15 | 0.61 |
| ACCS | -0.07 | -0.29 | 0.90 | -0.10 | 0.06 | -0.18 | 0.99 | 0.71 |
| LEPREL1 | 0.19 | -1.23 | 0.44 | -0.31 | 0.47 | -0.98 | 0.56 | -0.21 |
| LOC648057 | -0.14 | 0.04 | 0.62 | 0.36 | -1.82 | -0.81 | -0.24 | -0.53 |
| CLDN12 | -0.23 | -0.57 | 0.59 | -0.66 | 0.43 | 0.02 | 1.07 | 0.52 |
| KISS1R | -0.66 | -1.35 | 0.50 | 0.11 | -0.04 | -0.91 | 0.64 | 0.63 |
| C1orf116 | 0.13 | -0.71 | 0.82 | 0.08 | -0.15 | -0.62 | 1.02 | 0.43 |
| C11orf21 | -0.08 | -1.03 | 0.39 | -0.16 | -0.01 | -0.74 | 0.62 | 0.10 |
| CD37 | 0.17 | -0.89 | 0.74 | 0.39 | -0.28 | -0.28 | 0.44 | 0.06 |
| COLQ | -0.01 | 0.01 | 1.52 | 0.64 | 0.03 | -0.71 | 0.95 | 0.14 |
| ANGPTL6 | -0.49 | -0.29 | 0.90 | 0.45 | -0.48 | -0.73 | 0.77 | 0.41 |
| RARA | 0.06 | -0.30 | 0.74 | 0.11 | 0.18 | -0.24 | 0.82 | 0.05 |
| DPY19L3 | -0.05 | -0.07 | 0.55 | 0.00 | 0.20 | -0.45 | 0.38 | -0.21 |
| GAB3 | -0.14 | -0.46 | 0.68 | 0.25 | -0.14 | -0.07 | 1.09 | 0.41 |
| CORO1A | 0.27 | -0.12 | 1.50 | 0.42 | 0.28 | -0.10 | 1.23 | 0.29 |
| TACC1 | -0.25 | -0.36 | 0.42 | 0.14 | 0.31 | 0.12 | 0.69 | 0.42 |
| RAC2 | 0.07 | -0.59 | 0.75 | -0.03 | 0.39 | -0.25 | 0.70 | 0.06 |
| TELO2 | -0.52 | -1.83 | -0.21 | -0.27 | 0.47 | 0.21 | 0.84 | -0.49 |
| CCDC26 | 0.44 | -1.33 | 1.81 | 0.26 | 0.39 | -0.24 | 1.12 | -0.03 |
| EPOR | -0.18 | -1.08 | 0.73 | 0.52 | -0.24 | -0.86 | 0.63 | 0.18 |
| RASGRP4 | 0.14 | -0.47 | 1.05 | 0.66 | 0.07 | -0.37 | 0.89 | 0.32 |
| EFHC2 | -0.12 | -1.93 | 0.40 | -0.77 | 0.24 | -1.00 | 1.05 | 0.17 |
| NAT8B | 0.12 | -0.64 | 1.27 | 0.35 | 0.74 | -0.39 | 1.07 | -0.85 |
| RSPH3 | -0.02 | -3.04 | 1.02 | 0.02 | 0.79 | -0.21 | 1.00 | 0.67 |
| LGALS9 | -0.29 | -0.79 | 2.56 | 0.95 | 0.86 | -1.60 | 1.40 | 0.64 |
| IGFBP2 | 0.38 | -0.47 | 1.06 | 0.43 | 0.09 | -0.64 | 0.69 | 0.27 |
| GAB2 | 0.39 | -0.21 | 0.70 | -0.07 | 0.24 | -0.28 | 0.61 | 0.24 |
| FAM19A3 | -0.23 | -0.85 | 0.67 | 0.41 | -0.18 | -0.76 | 1.19 | 0.96 |
| OPTN | -0.10 | -0.72 | 0.91 | 0.35 | -0.48 | 0.04 | 1.21 | 0.04 |
| TXK | 0.10 | -2.47 | 1.22 | 0.79 | -0.24 | -0.42 | 1.80 | 1.12 |
| ODF2 | -0.02 | -0.41 | 0.44 | -0.57 | 0.67 | -0.16 | 1.05 | 0.59 |
| PLEKHA2 | -0.21 | -0.22 | 0.77 | 0.15 | 0.19 | -0.04 | 0.62 | 0.59 |
| TLR6 | 0.46 | -0.99 | 0.89 | -0.03 | 0.73 | 0.27 | 0.87 | -0.29 |
| PABPC1L | -0.13 | -0.14 | 0.38 | -0.40 | 0.45 | -0.33 | 0.92 | 0.31 |
| APOB48R | -0.21 | -0.93 | 1.07 | 0.57 | 0.23 | -0.72 | 0.83 | 0.65 |
| WASPIP | 0.20 | 0.06 | 1.07 | 0.51 | -0.54 | -0.68 | 0.08 | -0.31 |
| ST8SIA6 | 0.44 | -0.47 | 0.61 | 0.12 | 0.59 | 0.02 | 0.99 | 0.54 |
| C12orf76 | -0.21 | -1.11 | 0.37 | -0.11 | 0.25 | -0.15 | 0.94 | 0.11 |
| C12orf76 | -0.18 | -0.93 | 0.45 | -0.37 | 0.15 | -0.26 | 1.08 | 0.34 |
| APOBEC3B | -0.65 | -0.90 | 0.66 | 0.49 | 0.01 | -0.01 | 0.78 | 0.24 |
| TAGAP | -0.32 | -0.28 | 0.55 | 0.22 | 0.08 | 0.04 | 0.77 | 0.63 |
| CD37 | -0.05 | -1.34 | 1.10 | 0.05 | 0.43 | -0.37 | 1.00 | 0.76 |
| SMOX | 0.00 | -0.37 | 1.32 | 0.74 | -0.03 | 0.00 | 1.52 | 0.98 |
| PLAUR | -1.09 | 0.06 | 1.08 | 0.80 | -0.45 | -0.05 | 0.26 | 0.08 |
| SLA | 0.34 | -0.58 | 1.13 | -0.20 | 0.78 | -0.42 | 1.39 | 0.67 |
| PLCH1 | 1.02 | -0.22 | 1.17 | 0.54 | 0.04 | -0.70 | 0.40 | -0.70 |
| ZNF700 | -0.70 | 0.36 | 0.58 | 0.52 | -0.19 | -0.34 | 0.50 | -0.19 |
| IL18BP | -0.42 | -1.29 | 0.19 | -0.07 | 0.01 | -0.44 | 1.14 | 0.86 |
| PRIC285 | -0.11 | -0.68 | 0.57 | 0.37 | 0.23 | -0.06 | 0.98 | 0.65 |
| LCN2 | -0.51 | -0.13 | 1.45 | 1.03 | -0.16 | -0.19 | 1.00 | 0.63 |
| COL2A1 | -0.05 | -1.09 | 0.98 | 0.12 | -0.20 | -1.50 | 0.94 | 0.20 |
| CHN2 | -0.33 | -0.85 | 0.79 | -0.02 | 0.22 | 0.02 | 0.98 | 0.64 |
| IFI6 | -0.40 | -0.40 | 0.84 | 0.53 | -0.39 | -0.34 | 0.54 | 0.32 |
| RGS2 | -0.05 | 0.29 | 0.87 | 0.72 | -0.26 | -0.01 | 0.57 | 0.19 |
| MAPK13 | -0.41 | -1.22 | 1.02 | -1.52 | 0.38 | -0.57 | 0.76 | 0.32 |
| PTPRE | 0.38 | -0.30 | 2.13 | 0.57 | -0.02 | -0.97 | 2.17 | 0.52 |
| PTPRE | -0.20 | -0.55 | 1.66 | 0.48 | 0.49 | -0.87 | 1.44 | 0.63 |
| RBM20 | -1.04 | -1.11 | 0.93 | 0.29 | -1.00 | -0.47 | 0.48 | 0.34 |
| CD34 | 0.70 | -0.36 | 2.80 | 1.56 | 0.76 | -0.08 | 2.49 | 1.46 |
| SGK3 | -1.01 | -1.27 | -0.02 | -1.17 | 1.60 | 0.53 | 1.84 | 1.18 |
| ZNF225 | -1.18 | -0.27 | 0.36 | -0.08 | 0.08 | -0.29 | 0.32 | -0.27 |
| MS4A6A | -0.26 | -0.69 | 2.56 | 1.34 | 0.55 | -0.03 | 1.26 | 0.21 |
| MS4A6A | -0.25 | -0.26 | 2.50 | 1.36 | 0.25 | 0.13 | 1.18 | 0.24 |
| LOC151162 | -0.09 | -0.25 | 0.41 | 0.14 | 0.03 | -0.26 | 0.74 | 0.29 |
| RAB37 | -1.10 | -0.21 | 1.11 | 0.27 | 0.23 | 0.00 | 1.92 | 0.75 |
| RAB37 | -0.61 | -1.33 | 0.21 | 0.02 | 0.00 | -0.30 | 0.81 | 0.18 |
| TMPRSS11B | 0.04 | -0.76 | 0.51 | -0.45 | -0.03 | -0.74 | 0.59 | -0.38 |
| BMP1 | -0.08 | -0.95 | 0.80 | 0.71 | -0.07 | 0.00 | 0.65 | 0.62 |
| MAP4K1 | -0.36 | -0.94 | 0.71 | 0.50 | -0.22 | -0.49 | 0.75 | 0.59 |
| MAP4K1 | -0.46 | -0.55 | 0.59 | 0.51 | -0.53 | -0.41 | 0.63 | 0.46 |
| ACSL5 | -1.34 | 0.17 | 1.53 | 0.67 | -0.40 | 0.20 | 1.39 | 1.38 |
| PLCG2 | 0.10 | -0.67 | 0.61 | -0.12 | 0.00 | -0.27 | 0.83 | 0.40 |
| RNF166 | -0.57 | 0.07 | 0.28 | -0.23 | -0.02 | -0.24 | 0.49 | -0.12 |
| C14orf73 | -0.53 | -0.90 | 1.30 | 0.35 | -2.61 | -1.91 | 1.06 | 0.71 |
| KIAA1324L | -0.47 | -0.98 | 1.33 | 0.45 | 0.09 | -0.54 | 1.32 | 0.47 |
| CACNA2D4 | 0.21 | -1.13 | 1.55 | 0.02 | 0.48 | -0.85 | 1.12 | -0.02 |
| SMAD3 | -0.14 | -0.51 | 0.37 | -0.05 | 0.29 | -0.58 | 0.53 | 0.05 |
| GMFG | 0.21 | -0.13 | 0.96 | 0.58 | -0.18 | -0.25 | 0.64 | 0.11 |
| MRPS12 | 0.36 | -0.18 | 0.45 | 0.04 | 0.63 | 0.14 | 0.92 | -0.23 |
| FAM110A | -0.27 | -0.21 | 0.88 | 0.64 | -0.16 | -0.09 | 0.77 | 0.25 |
| ZNF438 | 0.02 | -0.06 | 0.75 | -0.06 | 0.45 | -0.23 | 0.79 | 0.25 |
| PRCP | 0.04 | -0.12 | 1.08 | 0.65 | -0.38 | -0.31 | 0.58 | -0.04 |
| IL8 | 0.93 | -0.22 | 2.49 | 0.69 | 0.02 | 0.48 | 1.18 | 0.07 |
| POU4F2 | 0.14 | -0.60 | 1.83 | 0.72 | 0.68 | -0.77 | 0.95 | -0.18 |
| FCER1A | -0.28 | -0.19 | 1.08 | 0.73 | 0.08 | 0.39 | 1.07 | 1.05 |
| NBPF22P | -0.30 | -0.07 | 0.93 | 0.04 | 0.15 | -1.03 | 0.62 | 0.03 |
| SLC35D3 | -0.02 | -1.65 | 1.20 | 0.42 | 0.02 | -0.30 | 0.81 | 0.46 |
| NBEA | -0.08 | -0.54 | 1.36 | 0.50 | 0.21 | -0.89 | 0.88 | -0.19 |
| ACBD7 | -0.35 | -1.09 | 0.52 | -0.06 | 0.41 | 0.15 | 1.60 | 1.32 |
| ACTA2 | 0.07 | -0.22 | 1.21 | 0.82 | 0.40 | -0.07 | 0.64 | 0.47 |
| ATP8B4 | -0.31 | -0.22 | 0.65 | 0.22 | 0.00 | 0.00 | 0.97 | 0.38 |
| ITGA11 | 0.03 | -0.94 | 1.07 | 0.46 | -0.20 | -0.80 | 1.35 | 0.98 |
| ITGAM | 0.55 | -0.27 | 2.11 | 1.29 | 0.05 | -0.09 | 1.21 | -0.05 |
| HSPA2 | 0.27 | 0.14 | 2.08 | 2.01 | -0.60 | -0.73 | 0.56 | 0.04 |
| CCBP2 | -0.62 | -1.09 | 0.55 | 0.11 | 0.01 | -0.61 | 0.44 | 0.01 |
| SUSD3 | 0.13 | -0.88 | 0.75 | -0.13 | 0.40 | -0.27 | 1.03 | 0.25 |
| TBXAS1 | -0.01 | -0.78 | 1.48 | 0.51 | 0.37 | -0.84 | 0.92 | 0.21 |
| MERTK | 0.01 | -0.03 | 0.50 | 0.29 | 0.11 | 0.15 | 0.56 | 0.27 |
| FYN | 0.31 | -0.79 | 0.58 | -0.27 | 0.56 | -0.65 | 0.74 | 0.45 |
| SPAG6 | 0.23 | -1.06 | 0.69 | -0.20 | 0.20 | -0.50 | 0.43 | -0.70 |
| LOC100129960 | 0.13 | 0.07 | 1.23 | 1.13 | -2.07 | -2.36 | -0.93 | -1.65 |
| RPP25 | -0.10 | -0.72 | 2.32 | 2.32 | -0.59 | -2.03 | 1.92 | 1.12 |
| C10orf54 | 0.44 | -0.18 | 1.79 | 0.02 | 0.86 | -0.08 | 1.74 | 0.86 |
| ME1 | 0.71 | -0.33 | 1.35 | 0.28 | -0.08 | -0.85 | 0.32 | -0.57 |
| CCR4 | -0.39 | -1.89 | 2.11 | 0.43 | 0.22 | 0.35 | 1.47 | 0.52 |
| LY6G6D | -0.14 | -1.04 | 0.19 | -0.01 | 0.40 | -0.87 | 0.79 | 0.61 |
| TMC8 | -0.44 | -0.23 | 0.66 | 0.53 | 0.07 | -0.11 | 0.96 | 0.63 |
| IDS | 0.01 | -0.49 | 0.78 | -0.20 | 0.44 | -0.01 | 0.76 | 0.03 |
| SDCCAG8 | -0.06 | -0.57 | 0.35 | -0.15 | 0.00 | 0.19 | 0.66 | 0.00 |
| MPL | -0.09 | -0.25 | 2.03 | 1.01 | -0.09 | -0.15 | 1.43 | 0.86 |
| C6orf115 | 0.17 | 0.21 | 1.08 | 0.87 | -0.08 | -0.62 | 0.51 | -0.01 |
| NKG7 | 0.01 | -0.11 | 2.05 | 0.81 | 0.25 | 0.31 | 1.25 | 0.75 |
| MAP4K2 | -0.21 | -0.13 | 0.83 | 0.40 | 0.08 | -0.08 | 0.87 | 0.47 |
| FXYD5 | -0.21 | -0.80 | 1.14 | 0.27 | 0.31 | -0.27 | 0.99 | 0.45 |
| RP2 | -0.53 | -1.07 | 1.36 | -0.25 | 0.31 | 0.62 | 1.38 | 1.01 |
| ABI2 | 0.67 | -0.14 | 0.89 | -0.10 | 0.26 | -0.71 | 0.73 | -0.22 |
| ARHGAP30 | -0.05 | -0.25 | 1.06 | 0.07 | 0.28 | 0.07 | 0.68 | 0.14 |
| GPR114 | 0.42 | -0.52 | 2.64 | 0.86 | -0.19 | 0.05 | 2.50 | 1.33 |
| TMEM54 | -0.78 | -0.15 | 0.84 | 0.46 | -0.88 | -0.88 | 1.03 | 0.69 |
| TSPAN4 | 0.35 | -0.38 | 1.53 | 1.17 | -0.12 | 0.69 | 1.12 | 0.74 |
| SMOX | -0.02 | -0.03 | 0.95 | 0.56 | -0.13 | -0.60 | 0.59 | 0.48 |
| HLA-A | -0.28 | -0.24 | 0.76 | 0.53 | -0.38 | -0.41 | 0.59 | 0.38 |
| LOC728975 | 0.15 | 0.68 | 0.95 | -0.08 | 0.61 | -0.37 | 1.00 | -0.43 |
| MSH4 | -0.33 | -0.14 | 1.46 | 0.00 | -0.66 | -1.06 | 1.12 | 0.23 |
| RASAL3 | 0.00 | -0.60 | 0.86 | 0.45 | 0.32 | -0.07 | 0.86 | 0.52 |
| EDG4 | -0.03 | -0.94 | 1.38 | 0.22 | 0.03 | -0.66 | 1.31 | 0.67 |
| CPXM1 | -0.23 | -0.39 | 0.80 | 0.51 | -0.63 | -0.24 | 0.61 | 0.21 |
| SUCNR1 | 0.26 | -0.30 | 1.83 | 1.02 | 0.19 | -0.94 | 1.39 | 0.07 |
| COLQ | 0.04 | -0.61 | 1.00 | 0.63 | -0.07 | 0.10 | 1.18 | 0.67 |
| CALCRL | 0.26 | -0.45 | 1.14 | 0.62 | 0.65 | 0.05 | 0.74 | 0.05 |
| RHOC | -0.49 | -0.59 | 0.60 | -0.06 | -0.25 | -0.71 | 0.89 | 0.21 |
| LPPR2 | -0.33 | -0.14 | 0.61 | 0.51 | 0.49 | -0.80 | 1.04 | 0.81 |
| TMEM45A | -0.60 | -0.36 | 0.37 | 0.03 | -0.68 | -0.25 | 0.33 | -0.77 |
| GAD1 | 0.34 | -0.22 | 0.53 | -0.10 | 0.36 | -0.18 | 0.62 | 0.27 |
| NYNRIN | -0.32 | -0.11 | 0.60 | 0.37 | -0.19 | -0.47 | 0.41 | 0.31 |
| SLC8A3 | 0.70 | -1.37 | 1.11 | -0.35 | 1.24 | -0.96 | 1.66 | 0.00 |
| S100A4 | -0.02 | -0.73 | 0.86 | 0.02 | 0.44 | -0.33 | 0.69 | -0.17 |
| TRIM10 | -0.53 | -0.39 | 0.39 | 0.00 | 0.00 | -0.53 | 0.66 | 0.51 |
| RGS19 | 0.08 | -0.22 | 0.76 | 0.36 | -0.10 | -0.47 | 0.51 | 0.00 |
| CYLN2 | 0.19 | -1.41 | 0.93 | 0.09 | 0.10 | -1.41 | 1.07 | 0.39 |
| LIMA1 | 0.29 | -0.66 | 0.38 | 0.01 | -0.13 | -0.15 | 0.06 | -0.77 |
| CASP8 | 0.03 | -0.57 | 1.19 | 0.40 | 0.04 | -0.03 | 0.66 | 0.16 |
| AEBP1 | -0.27 | -0.67 | 0.97 | 0.46 | -0.07 | -0.28 | 0.63 | 0.33 |
| NUDT14 | 0.15 | -0.91 | 0.87 | 0.21 | 0.09 | -0.72 | 0.35 | -0.01 |
| HIST1H2BC | -0.13 | -0.36 | 0.86 | 0.07 | -0.64 | -1.24 | 0.71 | 0.08 |
| LOC730517 | 0.02 | -0.02 | 1.10 | 0.10 | 0.43 | 0.18 | 0.68 | -0.02 |
| HIST1H2AG | 0.02 | -0.02 | 0.62 | -0.16 | -0.11 | -0.53 | 1.02 | 0.41 |
| ARHGEF6 | -0.10 | -0.09 | 0.72 | 0.25 | 0.14 | 0.10 | 0.70 | 0.27 |
| WDR26 | -0.30 | -0.52 | 0.65 | 0.06 | -0.59 | -0.64 | 0.60 | -0.05 |
| TERF1 | -0.16 | -0.23 | 0.40 | 0.23 | -0.09 | 0.02 | 0.87 | 0.48 |
| ETS1 | 0.33 | 0.06 | 1.50 | 1.30 | -1.25 | -1.08 | 0.30 | -0.06 |
| ARHGAP30 | 0.00 | -0.05 | 1.66 | 0.57 | 0.38 | 0.00 | 0.77 | 0.32 |
| HRC | -0.05 | -1.29 | 0.93 | -0.35 | 0.31 | -2.39 | 0.99 | 0.05 |
| DNAJB14 | 0.07 | -0.31 | 0.28 | -0.14 | -0.10 | -0.68 | 0.74 | -0.26 |
| ITPKA | -0.54 | -0.80 | 0.44 | 0.23 | -0.37 | -0.63 | 0.71 | 0.57 |
| MOBKL2C | -0.56 | -0.82 | 0.82 | 0.18 | -0.20 | -0.22 | 0.91 | 0.62 |
| VASN | -1.39 | -0.39 | 0.79 | 0.38 | -0.57 | -0.46 | 1.05 | 0.64 |
| ARG2 | -0.31 | -0.93 | 0.74 | 0.24 | 0.45 | -0.33 | 1.00 | 0.45 |
| CD2 | 0.30 | -1.04 | 0.58 | 0.03 | -0.97 | -0.19 | 0.53 | 0.40 |
| IL1B | 0.84 | -0.59 | 2.02 | 1.03 | 1.36 | -0.24 | 1.54 | 0.21 |
| MOV10 | -0.30 | -1.07 | 0.83 | 0.09 | 0.04 | -0.68 | 0.89 | 0.09 |
| FEM1B | 0.00 | -0.59 | 0.56 | -0.17 | 0.12 | -0.05 | 0.79 | -0.25 |
| DHTKD1 | -0.15 | -1.63 | 1.01 | 0.67 | -0.08 | -0.64 | 1.32 | 0.52 |
| USP36 | -0.08 | -1.03 | 0.01 | -0.98 | 0.46 | -0.74 | 0.62 | -0.01 |
| HIST2H2BE | -0.29 | -1.31 | 0.36 | 0.00 | -0.39 | -0.99 | 0.57 | 0.05 |
| SIRPA | -0.24 | -0.55 | 1.27 | 0.62 | 0.62 | 0.12 | 1.14 | 0.82 |
| CNFN | 0.09 | -0.08 | 0.56 | 0.44 | 0.19 | -0.17 | 0.43 | 0.18 |
| ADHFE1 | -0.86 | -1.05 | 1.10 | 0.49 | -0.09 | -0.61 | 1.27 | 0.87 |
| CLEC12A | -0.77 | -0.97 | 0.94 | 0.71 | 0.00 | -0.82 | 0.62 | 0.16 |
| TCTEX1D1 | -0.05 | -0.39 | 1.31 | -0.11 | 0.56 | -0.02 | 1.14 | 0.27 |
| CLIP2 | 0.00 | -0.98 | 0.18 | -0.16 | 0.04 | -0.39 | 0.82 | 0.22 |
| CDKN2B | -0.03 | -0.34 | 1.11 | 0.25 | 0.34 | -0.33 | 0.81 | 0.01 |
| TM6SF1 | -0.27 | -0.67 | 1.21 | 0.50 | -0.11 | -0.46 | 0.93 | 0.23 |
| HIST1H2BD | -0.46 | -0.99 | 0.42 | 0.32 | -0.18 | -0.69 | 0.91 | 0.43 |
| HIST1H2BD | -0.41 | -0.69 | 0.37 | 0.22 | -0.22 | -0.77 | 0.58 | 0.39 |
| LOC100129828 | 0.27 | -0.63 | 0.30 | 0.02 | 0.65 | -0.18 | 0.90 | -0.08 |
| PIK3CD | -0.10 | -0.37 | 0.41 | 0.09 | -0.05 | -0.19 | 0.58 | 0.01 |
| TACC2 | -0.60 | -0.86 | 0.18 | 0.06 | 0.46 | -0.10 | 0.74 | 0.48 |
| LOC654346 | 0.09 | 0.01 | 1.14 | 0.21 | -0.83 | -1.38 | 0.58 | 0.21 |
| FHOD3 | 0.22 | -1.11 | 1.91 | 0.28 | 0.45 | -1.96 | 1.84 | 0.20 |
| EMR4P | 0.06 | -0.06 | 1.34 | 0.48 | 0.73 | -0.12 | 1.83 | 0.82 |
| PDGFC | 0.20 | 0.01 | 0.43 | 0.22 | 0.06 | -0.57 | 0.63 | 0.24 |
| KATNAL1 | -1.05 | -0.68 | 0.49 | 0.12 | 0.42 | -0.11 | 0.71 | 0.01 |
| SLC7A8 | -0.75 | -0.72 | 0.80 | 0.34 | -0.82 | -0.70 | 0.68 | 0.63 |
| BST2 | 0.70 | -0.01 | 1.91 | 0.90 | 0.72 | 0.01 | 0.74 | -0.01 |
| TTC39B | 0.79 | -0.70 | 1.17 | 0.69 | 0.07 | -0.69 | 1.14 | -0.33 |
| CACHD1 | -0.50 | -0.14 | 1.08 | 0.68 | -0.94 | -0.43 | 1.26 | 0.53 |
| TMEM108 | 0.06 | -0.27 | 0.91 | 0.24 | 0.57 | -0.07 | 0.70 | 0.02 |
| RINL | -0.14 | -0.57 | 0.79 | 0.38 | 0.03 | -0.03 | 0.76 | 0.49 |
| NEU1 | -0.22 | -0.81 | 0.67 | 0.24 | 0.08 | -0.21 | 0.90 | 0.49 |
| TEK | -0.05 | -0.11 | 1.06 | 0.74 | 0.13 | -0.29 | 0.46 | 0.16 |
| ITGA5 | -0.28 | -0.45 | 1.00 | 0.32 | 0.00 | -0.35 | 0.78 | 0.45 |
| FES | -0.33 | -0.28 | 0.85 | 0.27 | -0.08 | 0.19 | 1.23 | 0.89 |
| LOC644150 | 0.05 | -0.56 | 0.53 | -0.05 | -0.18 | -0.51 | 0.50 | 0.32 |
| RGS18 | 0.24 | -0.47 | 1.56 | 0.22 | 0.52 | -0.17 | 1.17 | 0.17 |
| LOC652330 | -0.82 | -0.12 | 0.21 | 0.04 | 0.69 | -0.28 | 0.80 | 0.28 |
| MAN2B2 | -0.26 | -0.68 | 0.26 | -0.13 | 0.01 | -0.58 | 0.95 | 0.53 |
| FAM129A | -0.52 | -0.79 | 0.67 | 0.21 | 0.01 | 0.22 | 1.50 | 0.97 |
| PRDM1 | -0.44 | 0.01 | 1.70 | 0.59 | -0.57 | -1.05 | 1.08 | -0.53 |
| P2RX6 | -1.48 | -0.61 | 0.67 | 0.18 | -0.61 | -0.24 | 0.57 | 0.50 |
| AXUD1 | -0.16 | -0.70 | 1.12 | 0.01 | 0.19 | -0.53 | 0.98 | 0.29 |
| FAM102A | -0.75 | -1.61 | 0.41 | 0.14 | 0.08 | 0.33 | 0.77 | 0.66 |
| VAMP4 | 0.33 | -0.23 | 1.13 | -0.17 | -0.17 | 0.04 | 0.42 | -0.49 |
| FAM113B | -0.02 | -1.18 | 0.52 | 0.02 | 0.03 | -0.60 | 0.53 | -0.14 |
| ZNF680 | -0.06 | -0.30 | 0.74 | -0.13 | -0.08 | -0.45 | 0.79 | 0.28 |
| GRAMD2 | -0.05 | -0.31 | 0.75 | 0.02 | -0.02 | -0.58 | 0.97 | 0.48 |
| CSF3R | -1.09 | -0.08 | 0.85 | 0.21 | -0.65 | -0.98 | 1.13 | 0.97 |
| BIN1 | 0.07 | -0.52 | 0.50 | 0.29 | 0.18 | -0.44 | 0.80 | 0.52 |
| OTOF | 0.35 | -0.02 | 0.92 | 0.32 | 0.42 | 0.05 | 0.65 | 0.02 |
| SRGN | -0.24 | -0.53 | 1.26 | 0.43 | 0.24 | -0.06 | 1.14 | 0.84 |
| SRGN | -0.22 | -0.59 | 0.75 | 0.49 | 0.03 | -0.03 | 1.09 | 0.82 |
| CNIH2 | 0.05 | -0.67 | 0.76 | 0.30 | 0.15 | -0.85 | 0.67 | -0.32 |
| UPK1A | 0.29 | -0.84 | 0.91 | -0.21 | 0.75 | 0.28 | 1.01 | -0.45 |
| FLNC | 0.73 | -0.32 | 1.80 | 0.92 | -0.24 | -0.88 | 1.01 | 0.54 |
| KCNK17 | 0.48 | -0.47 | 1.46 | -0.07 | 0.78 | -0.14 | 1.60 | 0.56 |
| ATP9A | 0.27 | -0.51 | 2.24 | 0.73 | 1.21 | 0.42 | 1.82 | 1.04 |
| SAMD14 | 0.57 | -0.10 | 1.47 | 0.62 | 0.63 | 0.12 | 0.80 | -0.02 |
| PARP14 | -1.83 | -0.42 | 0.47 | 0.28 | 0.00 | -0.01 | 0.87 | 0.28 |
| TYROBP | 0.27 | -0.44 | 1.22 | 0.48 | 0.10 | -0.50 | 0.78 | 0.16 |
| RGL2 | 0.14 | -0.69 | 0.54 | 0.01 | 0.16 | -0.10 | 0.24 | 0.18 |
| S100A10 | -0.45 | -0.63 | 0.76 | 0.15 | -0.02 | -0.42 | 0.59 | -0.03 |
| MFGE8 | -0.65 | -1.22 | 0.85 | 0.66 | -0.20 | -0.54 | 1.01 | 0.73 |
| CCDC92 | 0.23 | -0.91 | 0.26 | -0.12 | 0.80 | -0.10 | 1.01 | 0.69 |
| FAM69A | 0.17 | -0.77 | 0.17 | 0.10 | -0.23 | -0.58 | 1.25 | 1.01 |
| TNFSF15 | 0.01 | -0.03 | 0.94 | 0.63 | -0.32 | -0.36 | 0.11 | -0.79 |
| ELF4 | 0.15 | -0.58 | 0.36 | -0.16 | 0.20 | 0.06 | 0.52 | 0.18 |
| PHTF1 | 0.17 | -0.90 | 0.69 | -0.17 | 0.09 | -0.67 | 0.72 | -0.18 |
| JUN | 0.13 | -1.97 | 1.18 | -0.13 | 0.49 | -1.18 | 0.64 | -0.99 |
| IDUA | -0.21 | -0.89 | 0.22 | 0.14 | -0.43 | -0.22 | 0.86 | 0.46 |
| ACSL4 | 0.20 | 0.18 | 0.80 | 0.28 | -0.37 | -0.86 | 0.23 | -0.48 |
| SLC9A1 | -0.38 | -0.35 | 0.52 | 0.26 | -0.03 | -0.05 | 0.87 | 0.56 |
| ABHD8 | 0.01 | -0.49 | 0.79 | 0.17 | 0.01 | -0.60 | 0.78 | 0.20 |
| BRP44 | -0.07 | -0.49 | 0.83 | 0.14 | -0.03 | -0.27 | 0.78 | 0.46 |
| FOLR1 | -0.53 | -0.97 | 0.06 | 0.01 | -0.11 | -0.58 | 0.54 | 0.43 |
| OAZ2 | 0.33 | -0.32 | 0.38 | -0.25 | 0.12 | 0.79 | 1.18 | 0.56 |
| KIAA1881 | 0.04 | -0.04 | 0.32 | -1.04 | -0.42 | -1.14 | 0.18 | 0.11 |
| MGC33556 | -0.02 | -0.41 | 1.36 | 0.82 | 0.28 | -0.36 | 0.91 | 0.07 |
| WNT11 | 0.54 | -0.38 | 1.92 | 0.37 | 0.59 | -0.53 | 1.33 | 0.06 |
| SHC1 | -0.16 | -0.64 | 0.64 | 0.17 | 0.02 | -0.38 | 0.95 | 0.34 |
| LOC653075 | -0.41 | -0.45 | 0.45 | 0.00 | -0.18 | 0.40 | 1.34 | 1.14 |
| KDELR3 | -0.94 | -1.66 | 0.71 | 0.65 | -0.19 | 0.08 | 0.56 | 0.56 |
| ACOT11 | -0.20 | -0.83 | 1.37 | 0.51 | 0.01 | -0.01 | 0.87 | 0.55 |
| CDRT4 | 0.03 | -1.13 | 0.46 | -0.04 | 0.37 | -0.51 | 0.76 | -0.02 |
| ISG20 | -0.02 | -0.98 | 1.53 | 0.25 | 0.28 | -0.88 | 1.08 | -0.08 |
| RTN1 | -0.32 | -0.17 | 0.96 | 0.54 | 0.26 | -0.10 | 1.03 | 0.41 |
| FYB | 0.36 | -0.36 | 1.57 | 0.47 | 0.11 | -0.74 | 0.98 | -0.13 |
| SPI1 | 0.15 | -0.03 | 1.23 | 0.41 | -0.22 | -0.46 | 0.50 | -0.32 |
| CCM2 | 0.52 | -0.07 | 1.74 | 0.93 | 0.84 | 0.20 | 1.45 | -0.16 |
| TEK | -0.02 | -0.19 | 1.20 | 0.94 | 0.15 | -0.08 | 0.63 | 0.02 |
| SCGN | -0.46 | -1.46 | 1.72 | 0.36 | -0.07 | -0.34 | 1.62 | 0.81 |
| SDC4 | -1.83 | -1.04 | 0.16 | -0.30 | -0.40 | -0.21 | 0.92 | 0.56 |
| JUP | -0.12 | -0.39 | 0.64 | 0.54 | 0.03 | -0.23 | 0.71 | -0.22 |
| LST1 | 0.10 | -0.85 | 2.42 | 0.68 | 0.07 | -1.03 | 1.75 | 0.30 |
| TRAF5 | -0.62 | -0.68 | 0.31 | 0.04 | -0.23 | -0.72 | 0.45 | 0.39 |
| SRC | 0.20 | -0.48 | 0.89 | 0.06 | -0.77 | -0.92 | 0.57 | -0.24 |
| ZNF93 | 0.13 | -1.90 | 1.26 | 0.16 | -0.34 | -1.42 | 1.23 | 0.62 |
| ZNF93 | 0.31 | -1.08 | 0.62 | 0.06 | -0.15 | -0.75 | 0.53 | 0.07 |
| GSTM4 | 0.04 | -0.46 | 0.60 | 0.22 | 0.01 | -0.35 | 0.56 | 0.29 |
| FERMT3 | -0.02 | -0.37 | 0.59 | 0.23 | 0.02 | -0.37 | 0.76 | 0.36 |
| FERMT3 | -0.25 | -0.17 | 1.00 | 0.40 | 0.24 | 0.03 | 1.16 | 0.66 |
| SFRP5 | -0.03 | -0.99 | 1.01 | 0.67 | 0.12 | -1.63 | 0.95 | 0.51 |
| TP53INP1 | -0.37 | -0.62 | 1.10 | 0.11 | -0.57 | -0.75 | 0.99 | 0.12 |
| TP53INP1 | -0.33 | -1.83 | 2.29 | 0.16 | -0.59 | -2.07 | 1.87 | 0.17 |
| CLCNKA | -1.31 | -1.92 | 1.73 | 0.67 | -0.05 | -0.60 | 2.37 | 1.25 |
| SIGLEC14 | -0.30 | -0.40 | 1.82 | 0.80 | 0.07 | -0.18 | 1.12 | 0.75 |
| CD82 | -0.05 | -0.21 | 1.07 | 0.01 | 0.35 | -0.01 | 0.98 | 0.09 |
| SUZ12P | 0.42 | -0.75 | 0.67 | -0.16 | -1.25 | -0.48 | 0.67 | -0.44 |
| AIF1 | 0.16 | -0.55 | 1.96 | 0.55 | 0.17 | -0.20 | 1.64 | 0.34 |
| NCKAP1L | 0.00 | -0.55 | 0.51 | 0.11 | 0.28 | -0.13 | 0.60 | 0.34 |
| HSPA6 | -0.74 | -0.09 | 1.95 | 0.88 | -0.72 | 0.14 | 1.05 | 0.84 |
| PPHLN1 | -0.08 | 0.21 | 0.99 | 0.63 | -3.09 | -1.99 | -0.73 | -1.22 |
| TMEM44 | -0.33 | -0.98 | 0.31 | -0.22 | 0.69 | 0.12 | 0.98 | -0.57 |
| PLXNB2 | -0.19 | -0.71 | 0.68 | 0.22 | 0.05 | -0.96 | 0.48 | 0.08 |
| LOC100129536 | -0.46 | -1.79 | 0.82 | -0.10 | -0.79 | -3.00 | 1.39 | 0.77 |
| SYNJ1 | -0.87 | -0.35 | 0.66 | 0.35 | -0.65 | -0.41 | 0.15 | -0.05 |
| LOC650759 | -0.47 | 0.23 | 0.60 | -0.06 | -0.11 | -0.83 | 0.08 | -0.28 |
| ZFAND3 | -0.21 | -1.15 | 0.42 | -0.06 | 0.71 | 0.23 | 0.88 | 0.24 |
| CD44 | 0.07 | -0.13 | 1.26 | 0.51 | -0.37 | -0.46 | 0.74 | 0.18 |
| C9orf85 | -0.29 | -0.46 | 0.27 | -0.44 | 0.23 | -0.04 | 0.55 | 0.01 |
| CMTM5 | -0.18 | -0.70 | 1.45 | 0.49 | 0.42 | -0.08 | 1.46 | 0.39 |
| ZNF493 | 0.08 | -1.31 | 0.81 | -0.25 | -0.05 | 0.01 | 1.27 | 0.85 |
| N4BP2L1 | -0.49 | -0.19 | 0.60 | 0.04 | 0.00 | -0.64 | 0.94 | 0.33 |
| N4BP2L1 | -0.78 | -2.45 | 0.78 | 0.41 | 0.22 | -0.29 | 0.71 | 0.30 |
| RSAD2 | 0.19 | -0.15 | 0.43 | 0.40 | -0.86 | -0.70 | 0.77 | -0.39 |
| LITAF | -0.27 | -0.69 | 1.70 | 1.41 | -0.02 | 0.02 | 1.10 | 1.02 |
| ANPEP | -0.34 | -0.65 | 1.23 | 0.69 | -0.23 | -0.27 | 0.76 | 0.26 |
| MR1 | -0.12 | -0.92 | 0.82 | -0.01 | 0.01 | -0.31 | 1.35 | 0.45 |
| PLVAP | -0.23 | 0.12 | 1.34 | 1.21 | -0.63 | -0.20 | 0.25 | 0.21 |
| SEMA4F | -0.07 | -0.47 | 0.55 | 0.18 | 0.23 | 0.02 | 0.54 | 0.48 |
| ITGA2B | 0.06 | -0.56 | 0.99 | 0.27 | -0.24 | -0.38 | 0.81 | 0.45 |
| SAMD9 | -0.19 | -0.88 | 0.83 | 0.18 | 0.16 | -0.72 | 1.58 | 1.02 |
| RASGRP2 | -0.38 | -0.94 | 0.93 | 0.40 | 0.46 | -0.03 | 0.86 | 0.42 |
| KCNV1 | 0.08 | -0.66 | 0.71 | 0.15 | -0.83 | -0.78 | 0.70 | -0.08 |
| IFI6 | -0.10 | -0.91 | 1.21 | 1.18 | -0.39 | -0.18 | 0.79 | 0.60 |
| CDH23 | -0.77 | -1.29 | 1.78 | 0.95 | 0.02 | -0.86 | 1.28 | 0.36 |
| TRPM4 | -0.12 | -0.66 | 1.43 | 0.56 | -0.04 | -0.18 | 1.38 | 0.74 |
| MYCN | 0.03 | -0.28 | 1.24 | -0.14 | 0.55 | -0.03 | 1.59 | 0.90 |
| SPATS2 | 0.03 | -0.16 | 0.59 | 0.26 | 0.27 | -0.18 | 0.72 | -0.08 |
| LOC728343 | -0.22 | -0.06 | 0.94 | 0.56 | -1.29 | -2.28 | 0.60 | 0.07 |
| HCST | 0.01 | -0.42 | 1.53 | 0.57 | 0.00 | -0.35 | 0.79 | 0.07 |
| CD63 | -0.04 | -0.29 | 0.74 | -0.15 | 0.41 | -0.54 | 0.92 | 0.04 |
| SVOPL | -0.06 | -0.83 | 1.13 | 0.39 | 0.50 | 0.48 | 1.18 | 0.56 |
| F8 | -0.57 | -2.79 | 0.11 | -0.01 | 0.36 | -1.50 | 0.42 | 0.01 |
| C1orf63 | -0.05 | -0.64 | 0.63 | -0.32 | 0.20 | -0.45 | 0.69 | 0.00 |
| LRGUK | -1.06 | -0.28 | 0.89 | -0.08 | 0.12 | -0.41 | 0.69 | 0.12 |
| ITGB3 | 0.16 | -1.94 | 2.08 | 0.73 | -1.67 | -1.44 | 1.48 | 1.36 |
| DHRS9 | 0.49 | -0.48 | 2.90 | 1.25 | 1.06 | 0.36 | 2.04 | 0.90 |
| CFH | -0.10 | -1.08 | 0.58 | -0.26 | 0.87 | -0.12 | 1.57 | 0.45 |
| CFH | -0.03 | -0.43 | 0.69 | -0.03 | 0.60 | -0.11 | 1.06 | 0.53 |
| CFH | 0.03 | -1.14 | 0.73 | -0.26 | 0.84 | -0.03 | 1.58 | 0.46 |
| CCL23 | -0.10 | -1.33 | 3.14 | 0.82 | 0.40 | -0.45 | 2.65 | 1.48 |
| TRIM67 | -0.75 | -0.86 | 2.56 | 1.09 | 1.33 | 0.25 | 2.13 | -0.25 |
| PDE4A | 0.05 | -0.68 | 0.53 | 0.05 | -0.16 | 0.14 | 0.39 | -0.26 |
| HIST1H3B | -0.01 | -0.15 | 0.31 | 0.13 | -0.18 | -0.56 | 0.88 | 0.63 |
| FAM43A | -0.37 | 0.04 | 1.13 | 0.52 | -0.02 | 0.17 | 0.69 | 0.53 |
| LOC731052 | 0.04 | -0.93 | 1.70 | 0.08 | 0.29 | -0.97 | 1.06 | -0.10 |
| SLC44A1 | 0.11 | -0.50 | 0.57 | -0.01 | 0.39 | -0.13 | 0.65 | -0.07 |
| VAV1 | 0.01 | -0.42 | 0.91 | 0.39 | 0.13 | -0.17 | 0.62 | 0.49 |
| ISG15 | 0.12 | -1.09 | 0.47 | 0.02 | 0.37 | -0.71 | 0.42 | -0.02 |
| PLEKHO1 | 0.01 | -0.31 | 0.89 | -0.26 | 0.19 | -0.31 | 0.86 | 0.13 |
| SOX13 | -0.09 | -0.39 | 0.39 | 0.00 | 0.02 | -0.32 | 0.66 | 0.38 |
| GRIN2C | 0.01 | -0.70 | 0.23 | -1.19 | 0.50 | -0.36 | 1.25 | 0.72 |
| FHL2 | 0.00 | -0.46 | 0.98 | 0.25 | 0.10 | -0.23 | 0.74 | 0.25 |
| ZNF681 | 0.36 | -0.39 | 1.26 | 0.60 | -1.74 | -3.24 | -0.76 | -1.00 |
| MGC39900 | 0.15 | -1.17 | 1.69 | 0.70 | 0.21 | -0.60 | 0.57 | 0.19 |
| ZNF274 | -0.46 | -0.82 | 0.84 | 0.14 | 0.27 | 0.15 | 0.93 | 0.45 |
| RHOC | -0.12 | -0.84 | 0.63 | 0.15 | -0.15 | -0.60 | 0.82 | 0.18 |
| MYO1F | -0.78 | -0.78 | 0.84 | 0.74 | 0.05 | -0.50 | 0.77 | 0.12 |
| TRIM63 | -0.17 | -1.42 | 1.53 | 0.61 | 0.16 | -0.21 | 0.77 | 0.31 |
| SIGLEC10 | 0.29 | -0.47 | 2.11 | 0.76 | 0.72 | -0.07 | 1.41 | 0.83 |
| IFIT3 | -0.45 | -1.12 | 1.04 | 0.09 | 0.41 | -0.27 | 1.28 | 0.34 |
| DENND5B | -0.48 | -1.19 | 0.33 | 0.18 | 0.05 | -0.51 | 0.77 | -0.39 |
| TUBB4 | -0.11 | -0.14 | 0.80 | 0.56 | -0.40 | -0.55 | 0.50 | 0.31 |
| FBXO32 | -0.61 | -0.50 | 0.30 | 0.03 | -0.58 | -0.03 | 0.56 | 0.51 |
| DSE | 0.01 | -0.64 | 0.55 | 0.36 | 0.03 | -0.53 | 0.50 | -0.01 |
| HBE1 | -0.97 | -1.36 | 0.68 | 0.43 | -0.47 | -0.80 | 0.56 | 0.51 |
| ARHGAP9 | 0.18 | -0.30 | 2.14 | 0.72 | 0.56 | -0.38 | 1.57 | 0.29 |
| ARAP3 | -0.43 | -0.62 | 1.26 | 0.26 | 0.13 | -0.32 | 1.36 | 0.63 |
| ARAP3 | -0.09 | -0.58 | 1.21 | 0.29 | 0.09 | -0.35 | 1.28 | 0.40 |
| CD3D | 0.06 | -0.06 | 1.85 | 0.89 | 0.32 | 0.16 | 1.15 | 0.65 |
| LBA1 | -0.06 | -0.57 | 1.22 | 0.99 | -0.19 | -0.27 | 0.65 | 0.13 |
| DHDH | -0.26 | -1.29 | 1.07 | 0.09 | -0.26 | -2.19 | 1.38 | 0.34 |
| ACSM3 | -0.13 | -0.37 | 0.90 | 0.85 | -0.17 | -0.48 | 0.34 | 0.20 |
| KIAA1370 | -0.14 | -0.28 | 1.01 | 0.69 | -2.37 | -0.98 | 0.22 | 0.02 |
| CDH26 | -0.18 | -0.52 | 0.62 | 0.01 | 0.06 | -0.12 | 0.93 | 0.68 |
| TESK2 | -0.23 | -1.29 | 0.45 | -0.23 | 0.01 | -0.42 | 0.97 | 0.47 |
| LOC644615 | 0.24 | -0.09 | 1.09 | 0.60 | -0.67 | -0.67 | 0.98 | 0.09 |
| IDH2 | -0.68 | -1.05 | 1.13 | 0.85 | -0.18 | -0.84 | 0.81 | 0.31 |
| GPR162 | -0.34 | -0.76 | 0.80 | 0.35 | 0.14 | -0.45 | 1.15 | 0.86 |
| RASGRP4 | -0.07 | -0.69 | 1.03 | 0.54 | 0.28 | -0.55 | 1.00 | 0.34 |
| SHANK3 | -0.12 | -0.08 | 1.22 | 0.73 | -0.38 | -0.29 | 0.98 | 0.59 |
| SHANK3 | -0.15 | -0.31 | 1.33 | 0.76 | -0.15 | -0.26 | 0.99 | 0.55 |
| ITGB2 | -0.44 | -0.46 | 0.50 | 0.18 | 0.08 | -0.03 | 0.85 | 0.60 |
| MMRN1 | 0.03 | -0.68 | 0.77 | 0.07 | 0.13 | -0.05 | 0.83 | 0.06 |
| SLC12A9 | 0.00 | -0.25 | 0.51 | 0.00 | 0.14 | 0.10 | 0.84 | 0.75 |
| SLC45A3 | -0.59 | 0.13 | 1.30 | 0.74 | -0.13 | 0.64 | 0.97 | 0.70 |
| EMR4 | -0.29 | -0.37 | 1.12 | 0.40 | 0.04 | -0.50 | 1.35 | 0.31 |
| GNAI2 | 0.21 | -0.40 | 0.59 | 0.36 | -0.37 | -0.61 | 0.49 | -0.03 |
| NRCAM | -0.64 | -1.27 | 0.61 | 0.01 | 0.55 | -0.02 | 0.63 | 0.24 |
| LOC650463 | 0.45 | -0.81 | 1.10 | -0.07 | 0.18 | -1.96 | 1.02 | 0.37 |
| NRIP3 | -0.52 | -0.50 | 0.39 | 0.18 | 0.17 | -0.25 | 0.96 | 0.76 |
| KIAA1370 | -0.59 | -0.59 | 0.48 | 0.02 | -0.72 | 0.04 | 0.54 | 0.31 |
| MAP1A | -0.24 | -2.05 | 0.94 | 0.50 | 0.34 | -0.68 | 0.63 | 0.32 |
| ENG | 0.10 | -0.14 | 1.62 | 1.00 | 0.43 | -0.01 | 0.98 | 0.65 |
| AIF1 | 0.13 | -0.67 | 1.19 | 0.44 | 0.36 | -0.52 | 1.42 | 0.38 |
| HTN3 | 0.87 | 0.29 | 1.99 | 0.90 | 0.75 | -0.03 | 0.90 | -0.09 |
| NDRG2 | -0.03 | -0.62 | 1.00 | 0.43 | 0.44 | -0.10 | 0.96 | 0.63 |
| OSBPL7 | -0.43 | -0.40 | 0.90 | 0.40 | -0.50 | -0.27 | 0.80 | 0.65 |
| HNMT | 0.12 | -0.66 | 1.76 | -0.24 | 0.44 | -0.27 | 1.54 | 0.27 |
| HNMT | 0.09 | -0.58 | 1.16 | -0.24 | 0.62 | -1.57 | 1.33 | 0.29 |
| CLDN10 | -1.27 | -0.44 | 0.58 | -0.46 | 0.07 | -0.62 | 1.37 | 0.78 |
| IL10RB | -0.30 | -0.09 | 0.62 | 0.24 | -0.01 | 0.02 | 0.75 | 0.66 |
| DDX25 | 0.62 | -0.81 | 0.63 | 0.40 | -0.03 | -0.42 | 0.42 | 0.17 |
| C17orf65 | -0.21 | -0.44 | 0.49 | 0.33 | -0.01 | -0.86 | 0.59 | 0.01 |
| SETX | -0.87 | -1.20 | -0.09 | -0.37 | 0.50 | -0.23 | 1.02 | 0.42 |
| SYTL3 | 0.08 | -0.22 | 1.25 | 0.31 | 0.14 | -0.42 | 1.17 | 0.60 |
| CASP4 | 0.05 | -1.11 | 0.33 | -0.13 | -0.16 | -0.35 | 0.75 | 0.34 |
| EMR2 | -0.33 | -0.99 | 1.07 | 0.30 | -0.03 | -0.23 | 1.26 | 0.79 |
| ADORA2A | 0.19 | -0.28 | 0.41 | -0.04 | 0.04 | 0.06 | 0.90 | -0.34 |
| SLA | 0.05 | -0.10 | 1.47 | 0.01 | 0.64 | -0.37 | 1.63 | 0.48 |
| C6orf150 | -0.61 | -0.80 | 0.35 | -0.09 | 0.08 | -0.08 | 0.60 | 0.34 |
| ITGA9 | -0.39 | -0.44 | 0.35 | 0.03 | 0.05 | 0.09 | 0.70 | 0.23 |
| NOTCH2NL | 0.31 | -1.02 | 0.84 | 0.30 | -0.04 | -0.16 | 0.37 | -0.45 |
| TBC1D10C | 0.39 | -0.63 | 1.86 | 1.08 | -0.51 | -0.31 | 1.56 | 1.06 |
| KYNU | 0.23 | -0.92 | 0.76 | -0.49 | 0.42 | -0.63 | 0.73 | -0.08 |
| BMP8B | -0.29 | -0.06 | 0.66 | 0.53 | -1.04 | -1.49 | 0.19 | -0.31 |
| APOBEC3F | -0.64 | -0.27 | 0.89 | 0.73 | 0.15 | -0.13 | 1.29 | 0.90 |
| PAPSS2 | -0.26 | -0.39 | 0.72 | 0.14 | 0.41 | 0.01 | 0.95 | 0.52 |
| LFNG | -0.19 | -1.96 | 2.56 | 1.44 | -0.05 | 0.05 | 1.79 | 0.77 |
| PTGS1 | 0.10 | -0.80 | 0.96 | 0.26 | -0.25 | -0.93 | 0.73 | 0.04 |
| UBR1 | 0.29 | -0.28 | 0.35 | 0.27 | 0.03 | 0.02 | 0.64 | 0.26 |
| PNPLA7 | -0.14 | -0.03 | 0.67 | 0.35 | -0.17 | -0.17 | 0.80 | 0.47 |
| P2RX1 | 0.02 | -0.41 | 0.34 | -0.06 | 0.07 | -0.60 | 0.50 | -0.07 |
| RYR1 | -0.22 | -1.43 | 0.98 | 0.24 | -0.36 | -1.43 | 0.90 | -0.01 |
| RYR1 | -0.38 | -0.95 | 0.63 | 0.14 | -0.52 | -1.10 | 0.55 | -0.35 |
| FAM101B | 0.41 | -0.45 | 1.55 | 0.41 | -0.01 | -0.11 | 0.80 | 0.23 |
| MMP23B | -0.14 | -0.94 | 0.79 | -0.14 | 0.76 | -0.36 | 1.33 | 0.82 |
| PELI1 | -0.23 | -0.16 | 0.73 | 0.63 | 0.15 | 0.04 | 0.77 | 0.29 |
| SPRY4 | -0.08 | -0.61 | 1.21 | 0.92 | 0.65 | 0.08 | 0.68 | 0.34 |
| COL7A1 | 0.18 | -0.38 | 0.95 | -0.51 | 0.04 | -0.62 | 0.67 | -0.22 |
| C1QTNF6 | -0.06 | -0.57 | 0.82 | 0.65 | -0.19 | 0.02 | 0.99 | 0.81 |
| CD86 | -0.01 | -0.11 | 0.92 | 0.23 | -0.07 | -0.13 | 0.43 | -0.15 |
| TICAM2 | -1.86 | -0.20 | 0.02 | -0.60 | 0.73 | -0.02 | 1.05 | -0.05 |
| HIST1H2BE | 0.10 | -0.23 | 0.68 | 0.36 | -0.87 | -2.16 | 0.37 | -0.56 |
| EMR4 | -1.85 | -0.05 | 1.09 | 0.38 | -0.21 | -0.36 | 1.38 | 0.60 |
| PSMB8 | -0.92 | -0.93 | 0.64 | 0.58 | -0.99 | -0.25 | 1.19 | 0.54 |
| CYTH1 | -0.51 | -0.30 | 0.71 | 0.20 | 0.09 | -0.18 | 0.94 | 0.79 |
| CSF1R | 0.11 | -1.41 | 3.05 | 0.90 | 0.22 | -0.47 | 2.66 | 0.94 |
| SLAMF6 | -0.40 | -0.76 | 1.51 | 0.96 | -0.16 | -0.46 | 1.63 | 1.01 |
| SYN1 | -0.06 | -1.03 | 0.32 | -0.08 | 0.18 | -0.36 | 0.54 | 0.08 |
| C9orf16 | -0.05 | 0.05 | 1.13 | 0.36 | -0.13 | -0.44 | 0.35 | -0.06 |
| KIAA1539 | -0.30 | -0.57 | 1.16 | 0.42 | -0.49 | -0.69 | 0.83 | 0.07 |
| WIPI1 | -0.17 | -1.59 | 0.95 | 0.17 | 0.18 | -0.72 | 1.28 | 0.31 |
| TIE1 | -0.07 | -0.37 | 1.31 | 0.49 | 0.13 | 0.07 | 0.89 | 0.47 |
| AHR | -0.48 | -0.18 | 0.53 | -0.38 | -0.31 | -0.21 | 0.44 | -0.92 |
| PLEKHM1 | -0.01 | -1.33 | 0.32 | -0.34 | -0.21 | 0.14 | 1.26 | -0.06 |
| SLAMF6 | -0.52 | -0.28 | 1.06 | 0.31 | -0.08 | -0.10 | 1.34 | 0.85 |
| NXF3 | -0.49 | -0.52 | 0.58 | 0.11 | -0.17 | -0.21 | 0.91 | 0.62 |
| YPEL3 | 0.09 | -0.35 | 0.88 | 0.47 | -0.52 | -0.68 | 0.36 | 0.04 |
| PADI4 | -0.75 | -0.87 | 1.83 | 0.61 | -0.49 | -0.81 | 1.63 | 0.83 |
| LMTK3 | 0.02 | -0.61 | 0.70 | -0.19 | 0.09 | -0.64 | 0.84 | 0.57 |
| KIT | 0.00 | -0.18 | 0.98 | 0.50 | -0.12 | 0.17 | 0.74 | 0.67 |
| PTRF | -0.20 | -0.61 | 0.43 | 0.15 | 0.08 | -0.08 | 0.73 | 0.65 |
| MAP3K8 | -0.18 | -1.06 | 0.52 | -0.33 | 0.38 | -0.40 | 0.81 | 0.01 |
| PSCDBP | 0.00 | 0.19 | 1.82 | 0.97 | 0.00 | 0.14 | 0.84 | -0.14 |
| ADORA3 | -0.01 | -0.38 | 1.87 | 0.23 | 0.26 | -0.15 | 1.61 | 0.33 |
| IL18RAP | 0.28 | -0.72 | 1.52 | -0.07 | 0.55 | -0.20 | 1.02 | 0.07 |
| GABRE | 0.37 | 0.00 | 0.71 | 0.51 | -0.23 | -0.55 | 0.37 | -0.14 |
| RHBDL1 | -0.34 | -0.53 | 0.50 | -0.20 | 0.48 | -0.58 | 0.54 | -0.19 |
| HBD | -0.18 | -0.89 | 0.13 | -0.21 | -0.15 | -0.58 | 0.89 | 0.69 |
| OSBPL10 | -1.65 | -2.75 | 0.22 | 0.13 | -2.39 | -1.38 | 0.62 | 0.28 |
| PRKCB1 | 0.04 | -0.11 | 0.84 | 0.68 | 0.24 | 0.34 | 0.95 | 0.45 |
| STAP1 | -0.01 | -0.09 | 0.69 | 0.10 | 0.22 | -0.05 | 0.49 | -0.22 |
| GNB5 | -1.00 | -0.77 | 0.34 | 0.11 | 0.70 | 0.32 | 1.51 | 0.86 |
| SCAMP5 | -1.01 | -2.06 | 1.16 | 0.48 | 0.15 | -0.94 | 1.07 | 0.64 |
| ITGB5 | 0.11 | -0.61 | 1.20 | 0.05 | -0.02 | -0.46 | 0.89 | 0.32 |
| ITGB5 | -0.08 | -0.79 | 0.73 | 0.06 | 0.09 | -0.35 | 1.08 | 0.36 |
| LPAR2 | -0.35 | -0.34 | 0.73 | -0.06 | 0.39 | -0.62 | 0.90 | 0.21 |
| FLJ16323 | -0.34 | -0.54 | 0.33 | 0.13 | -0.32 | -0.66 | 0.32 | 0.16 |
| HSPA2 | -0.31 | -0.13 | 1.90 | 1.80 | -0.01 | -0.06 | 0.88 | 0.82 |
| TLR4 | 0.26 | -0.28 | 0.86 | 0.75 | 0.36 | -0.41 | 0.59 | -0.10 |
| HMOX1 | -0.27 | -1.02 | 1.59 | 0.64 | 0.06 | -1.45 | 1.15 | -0.89 |
| C1orf54 | -0.46 | -0.26 | 0.49 | -0.03 | 0.33 | 0.03 | 0.80 | 0.70 |
| RHBDF1 | -0.43 | -0.76 | 0.96 | 0.31 | -0.65 | -0.51 | 1.45 | 1.09 |
| HYAL3 | -0.11 | -0.85 | 0.79 | 0.01 | 0.10 | -0.66 | 0.72 | -0.01 |
| FBXL13 | 0.83 | -1.18 | 1.28 | 0.01 | 0.70 | -0.76 | 1.24 | -0.40 |
| PITPNM1 | -0.21 | -0.29 | 1.07 | 0.97 | -0.43 | -0.42 | 0.76 | 0.62 |
| NHLRC3 | -0.38 | -0.09 | 0.86 | 0.58 | -1.70 | -0.34 | 0.49 | -0.05 |
| NLRC3 | 0.03 | -0.03 | 1.37 | 0.56 | 0.19 | -0.54 | 1.25 | 0.81 |
| KCNK6 | -0.38 | -0.38 | 0.90 | 0.18 | -0.11 | -0.24 | 0.72 | 0.39 |
| HSDL1 | 0.05 | -1.52 | 0.83 | -0.11 | -0.02 | -0.03 | 1.28 | 0.16 |
| MS4A3 | -0.12 | -0.47 | 1.14 | 0.85 | 0.13 | 0.03 | 0.78 | 0.12 |
| YPEL5 | -0.39 | -0.63 | 0.69 | 0.46 | -0.09 | -0.31 | 1.23 | 0.64 |
| CCRL2 | -0.19 | -0.39 | 1.03 | 0.39 | 0.08 | -0.13 | 1.08 | 0.65 |
| SLC25A29 | -0.17 | -0.36 | 0.87 | 0.26 | -0.59 | -0.71 | 0.91 | 0.50 |
| MAP7 | -0.12 | -0.46 | 1.18 | 0.38 | -0.52 | -0.30 | 0.95 | -0.17 |
| MXRA7 | -0.14 | -0.56 | 0.68 | 0.00 | 0.57 | -0.01 | 1.02 | 0.80 |
| CRYAB | -0.08 | -1.12 | -0.04 | -0.47 | 0.54 | -0.78 | 0.74 | -0.67 |
| VASH1 | 0.23 | -0.89 | 0.47 | -0.01 | 0.20 | -0.89 | 0.43 | -0.08 |
| TUBB2B | -0.33 | -0.89 | 0.41 | 0.18 | -0.01 | -0.70 | 0.74 | 0.68 |
| CENTA1 | -0.09 | -0.16 | 0.43 | 0.15 | 0.34 | 0.28 | 0.88 | 0.66 |
| GPR56 | 0.07 | -1.30 | 1.79 | 0.65 | 0.03 | -0.98 | 1.98 | 0.50 |
| MMD | -0.16 | -0.79 | 1.03 | 0.04 | -0.03 | -0.52 | 0.49 | -0.36 |
| CTRC | -0.35 | 0.02 | 0.13 | -0.22 | 0.07 | 0.15 | 0.60 | -0.87 |
| PMAIP1 | 0.16 | -0.36 | 0.94 | 0.19 | -0.75 | -1.24 | 0.05 | -0.68 |
| LOC650919 | 0.08 | -0.08 | 0.77 | 0.73 | -0.46 | -2.60 | 0.92 | 0.35 |
| PLEKHG3 | 0.31 | -0.14 | 1.20 | 0.32 | 0.29 | 0.10 | 1.09 | 0.34 |
| IFIT2 | -0.19 | -0.85 | 0.78 | -0.12 | 0.22 | -0.59 | 0.75 | -0.30 |
| TNNT1 | 0.12 | -0.65 | 1.13 | 0.48 | -0.52 | -0.74 | 0.43 | 0.00 |
| GARNL4 | -0.46 | -0.06 | 0.84 | 0.19 | 0.18 | 0.13 | 1.02 | 0.77 |
| C7orf58 | -0.51 | -1.00 | 0.67 | 0.60 | -0.61 | -0.55 | 0.78 | -0.02 |
| ZNF430 | 0.63 | 0.23 | 0.89 | 0.33 | -0.63 | -0.92 | 0.17 | -0.04 |
| LASS5 | -0.05 | -0.54 | 0.23 | 0.20 | 0.05 | -0.20 | 0.74 | 0.54 |
| CD69 | 0.45 | -0.78 | 1.08 | 0.56 | -0.42 | -1.63 | 0.44 | -0.97 |
| NBL1 | -0.37 | -0.83 | 0.73 | 0.38 | -0.14 | -0.67 | 0.53 | 0.23 |
| ENDOD1 | -0.41 | -0.41 | 0.72 | 0.18 | -0.50 | -0.11 | 0.52 | 0.31 |
| C1RL | -0.24 | -0.97 | 0.64 | 0.03 | -0.28 | -0.85 | 0.88 | 0.41 |
| FAM116B | 0.10 | -0.27 | 0.81 | 0.20 | -0.05 | -0.41 | 0.73 | 0.30 |
| TRAF4 | 0.31 | -0.45 | 0.46 | 0.36 | -0.06 | 0.06 | 0.94 | -0.83 |
| ME3 | -0.46 | -0.76 | 1.42 | 0.78 | -0.26 | -1.39 | 1.10 | 0.80 |
| LOC650111 | -0.03 | -1.33 | 0.26 | 0.02 | 0.16 | -0.31 | 0.49 | 0.16 |
| DUSP5 | 0.10 | -0.27 | 1.38 | 0.56 | -0.02 | -0.21 | 0.76 | 0.29 |
| PLAC8 | 0.66 | -2.14 | 2.17 | -0.10 | 1.06 | -1.43 | 1.43 | -0.98 |
| PLAC8 | 0.67 | -0.83 | 1.77 | -0.13 | 1.05 | -0.21 | 1.29 | -0.28 |
| CD84 | 0.07 | -0.99 | 1.64 | 0.46 | 0.27 | -0.29 | 1.45 | 0.67 |
| STK17B | 0.00 | -0.33 | 1.01 | 0.35 | 0.45 | -0.25 | 0.87 | 0.36 |
| ADHFE1 | -0.75 | -0.35 | 0.79 | 0.17 | -0.83 | -0.61 | 0.84 | 0.61 |
| DPY19L2 | -0.03 | -0.43 | 0.87 | 0.09 | 0.18 | -0.54 | 0.95 | 0.39 |
| TIMP3 | 0.30 | -0.04 | 1.35 | 0.94 | 0.90 | -0.12 | 0.98 | 0.51 |
| SPI1 | 0.15 | -0.70 | 0.97 | 0.51 | -0.08 | -0.41 | 0.59 | -0.02 |
| FBXO32 | -1.13 | -0.28 | 0.59 | 0.35 | -0.82 | 0.11 | 1.04 | 0.28 |
| FCGRT | 0.03 | -0.64 | 1.04 | 0.36 | -0.04 | -0.63 | 0.69 | 0.48 |
| PRKCB | 0.14 | -0.08 | 0.64 | 0.40 | 0.06 | -0.16 | 0.49 | -0.01 |
| PRKCB | 0.03 | -0.27 | 0.66 | 0.30 | 0.04 | -0.23 | 0.47 | -0.01 |
| TMEM8 | 0.00 | -0.99 | 0.84 | 0.83 | 0.10 | 0.00 | 0.76 | 0.33 |
| TRNP1 | 0.22 | -0.73 | 0.70 | 0.02 | 0.50 | -0.16 | 0.88 | 0.67 |
| TG | 0.54 | 0.05 | 0.59 | -1.00 | 0.30 | -1.22 | 0.40 | -0.57 |
| TTLL3 | -0.21 | -0.84 | 0.52 | -0.02 | -0.37 | -1.05 | 0.65 | 0.23 |
| MAN2B1 | -0.13 | -0.23 | 0.34 | 0.23 | 0.29 | -0.32 | 0.44 | 0.43 |
| MATN2 | -0.46 | -0.58 | 0.49 | 0.36 | -0.57 | -0.88 | 0.51 | 0.34 |
| TOX2 | 0.00 | -0.23 | 1.11 | 0.66 | 0.29 | -0.30 | 0.44 | 0.36 |
| ARRB2 | 0.52 | -0.78 | 1.99 | 0.67 | 0.70 | -0.26 | 0.88 | -0.26 |
| CDKN2B | 0.11 | -0.34 | 0.87 | 0.13 | 0.54 | -0.40 | 1.12 | 0.24 |
| ABCC3 | 0.02 | -1.27 | 2.78 | 1.04 | 0.32 | -0.86 | 2.22 | 0.96 |
| MYH9 | 0.10 | -0.36 | 0.49 | 0.36 | 0.00 | -0.49 | 0.64 | -0.05 |
| OR2B6 | 0.65 | -1.17 | 0.76 | 0.08 | 0.19 | -1.14 | 0.90 | 0.27 |
| CLCNKA | -0.97 | -1.59 | 1.81 | 0.56 | -0.35 | -0.60 | 2.01 | 1.38 |
| IFIT3 | -0.20 | -0.61 | 0.82 | 0.04 | 0.17 | -0.47 | 0.95 | 0.36 |
| CCL5 | -0.44 | -0.17 | 2.19 | 0.75 | 0.06 | -0.18 | 1.51 | 0.62 |
| CCL5 | -0.26 | -1.46 | 2.31 | 0.30 | 0.36 | -0.12 | 1.72 | 0.47 |
| BMP6 | 0.39 | -0.30 | 1.44 | 0.47 | 0.71 | -0.03 | 1.35 | 0.57 |
| OBFC2A | -0.12 | -0.45 | 0.96 | 0.21 | -0.16 | -0.38 | 0.89 | 0.10 |
| CD3D | 0.25 | -0.35 | 1.92 | 0.88 | 0.18 | 0.07 | 1.02 | 0.36 |
| ZYX | 0.19 | -0.05 | 0.88 | 0.32 | 0.12 | -0.29 | 0.55 | -0.14 |
| HLA-H | -0.23 | -0.46 | 0.66 | 0.09 | -0.67 | -0.10 | 0.16 | -0.21 |
| TUBA4A | 0.22 | -0.41 | 1.59 | 1.21 | -0.91 | -1.24 | 0.45 | 0.02 |
| PLXDC2 | 0.11 | -1.20 | 1.21 | 0.41 | -0.03 | -1.75 | 1.36 | 0.20 |
| SLC37A1 | -0.01 | -1.34 | 0.63 | 0.01 | 0.23 | -0.50 | 1.05 | 0.19 |
| LPXN | 0.11 | -0.40 | 1.55 | 0.54 | 0.04 | -0.48 | 0.90 | 0.18 |
| DPEP2 | -1.52 | -0.67 | 0.48 | 0.17 | 0.02 | -0.55 | 0.42 | 0.14 |
| MS4A3 | 0.12 | -0.20 | 1.54 | 0.96 | 0.13 | -0.54 | 0.85 | 0.00 |
| GPR37 | 0.16 | -0.61 | 0.89 | -0.41 | 0.19 | -0.01 | 1.82 | 0.26 |
| PANX2 | 0.09 | -1.41 | 1.74 | 0.92 | 0.33 | -2.86 | 0.74 | 0.33 |
| C20orf100 | -0.09 | -0.33 | 0.90 | 0.20 | 0.09 | 0.09 | 0.40 | 0.19 |
| IGFBP5 | -0.14 | -0.33 | 1.05 | 0.13 | -1.14 | -1.01 | 0.97 | 0.81 |
| PLCB2 | -0.14 | -0.48 | 0.99 | 0.31 | -0.04 | -0.46 | 1.05 | 0.60 |
| CDKN1A | -0.74 | -1.43 | 2.04 | 1.09 | -0.23 | -0.77 | 1.64 | 0.89 |
| SLC43A2 | -0.10 | -0.20 | 0.84 | 0.04 | 0.40 | -0.28 | 0.79 | 0.37 |
| PARVG | 0.64 | -0.05 | 2.06 | 0.92 | 0.97 | -0.06 | 1.56 | 0.78 |
| ARHGEF3 | -0.08 | -0.35 | 1.33 | 0.55 | -0.41 | 0.14 | 1.54 | 0.92 |
| DIAPH3 | -0.53 | -0.16 | 0.25 | -0.54 | 0.64 | 0.31 | 0.91 | 0.69 |
| PEAR1 | -0.01 | -0.19 | 1.05 | 0.95 | 0.01 | -0.47 | 0.64 | 0.04 |
| TPM4 | -0.26 | -0.53 | 0.76 | 0.02 | 0.16 | -0.34 | 0.98 | 0.41 |
| KIAA0913 | 0.07 | -0.43 | 0.55 | 0.11 | -0.14 | -0.38 | 0.82 | 0.52 |
| NFATC2IP | 0.13 | -0.77 | 0.47 | 0.22 | -1.46 | 0.01 | 0.32 | -0.02 |
| KRT72 | 0.18 | -0.18 | 1.65 | -0.34 | 0.38 | -0.73 | 1.21 | 0.31 |
| MTMR11 | -1.70 | -0.48 | 1.94 | 0.87 | -0.24 | 0.12 | 1.17 | 0.94 |
| ANXA3 | 0.00 | 0.00 | 1.57 | 1.05 | -0.41 | -0.85 | 0.55 | 0.11 |
| F2R | 0.11 | -0.07 | 1.20 | 0.60 | 0.25 | -0.14 | 1.05 | 0.44 |
| TRIM15 | -0.28 | -1.33 | -0.01 | -0.10 | 0.32 | 0.01 | 0.80 | 0.75 |
| TXNIP | -0.37 | -1.12 | -0.01 | -0.16 | -0.02 | -0.50 | 0.58 | 0.23 |
| RAB7L1 | -0.33 | -0.53 | 0.54 | 0.36 | -0.29 | -0.15 | 1.19 | 0.39 |
| BCL3 | 0.01 | 0.08 | 1.56 | 0.40 | -0.58 | -1.55 | 1.20 | -0.16 |
| FOXA2 | 0.78 | -1.04 | 1.41 | 0.24 | 0.99 | -0.13 | 1.34 | 0.02 |
| FSD2 | -0.73 | -1.13 | 0.13 | 0.06 | -0.07 | -0.85 | 1.51 | 1.26 |
| TNFRSF1B | -0.31 | -1.13 | 0.38 | -0.15 | -0.03 | -0.57 | 0.94 | 0.51 |
| MIB2 | 0.22 | -1.26 | 0.71 | -0.12 | 0.23 | -1.71 | 1.33 | 1.08 |
| CDH26 | -0.13 | -0.71 | 0.47 | 0.16 | 0.37 | 0.13 | 0.83 | 0.55 |
| CDH26 | 0.22 | -0.76 | 0.93 | 0.16 | 0.15 | -0.89 | 1.28 | 0.96 |
| ECM1 | -0.63 | -0.50 | 2.13 | 0.92 | -0.48 | -0.22 | 1.09 | 0.34 |
| ST6GAL1 | 0.10 | 0.00 | 0.88 | 0.37 | -1.24 | -0.84 | -0.03 | -0.37 |
| C1orf63 | 0.15 | -0.64 | 1.08 | -0.14 | -1.02 | -1.54 | -0.11 | -0.32 |
| KLHL3 | -1.68 | -0.08 | 1.21 | 0.57 | -0.62 | -0.13 | 1.48 | 1.07 |
| APBA2BP | 0.16 | -2.10 | 0.17 | 0.03 | 0.56 | -0.09 | 1.19 | 0.84 |
| UBE2H | -0.56 | 0.22 | 1.27 | 0.99 | -0.71 | -0.16 | 0.99 | 0.37 |
| INPP5B | -0.27 | 0.23 | 0.33 | -0.17 | 0.22 | 0.47 | 0.57 | 0.11 |
| ROR2 | -0.07 | -0.25 | 0.46 | 0.14 | 0.07 | 0.03 | 0.52 | 0.20 |
| RPL23AP13 | 0.48 | 0.38 | 0.85 | 0.21 | -0.02 | 0.02 | 1.08 | 0.95 |
| FHL2 | 0.18 | -0.23 | 1.06 | 0.34 | 0.00 | -0.33 | 0.91 | 0.23 |
| PPP1R16B | -0.59 | -0.01 | 1.10 | 0.42 | -0.26 | -0.09 | 0.97 | 0.70 |
| CDC42SE2 | 0.60 | 0.00 | 0.62 | -0.10 | -0.84 | -0.81 | 0.24 | -0.65 |
| FRAS1 | -0.10 | -0.68 | 1.30 | 0.01 | 0.13 | -0.82 | 1.12 | 0.41 |
| IL10RA | 0.52 | -0.76 | 1.88 | 0.84 | -0.08 | -1.02 | 0.87 | -0.52 |
| ZBTB48 | 0.01 | -0.57 | 0.76 | 0.25 | -0.16 | -0.20 | 0.57 | 0.17 |
| HPS5 | -0.67 | -0.36 | 0.40 | -0.55 | 0.22 | -0.65 | 0.38 | 0.25 |
| RALB | -0.09 | -0.09 | 0.75 | 0.28 | 0.27 | 0.07 | 0.64 | 0.36 |
| PIK3CG | -0.65 | 0.49 | 1.32 | 0.60 | -1.12 | -0.15 | 0.21 | 0.12 |
| TM4SF1 | -0.22 | -0.19 | 1.87 | 0.93 | 0.38 | 0.44 | 0.82 | 0.48 |
| H2AFY | -0.20 | -0.67 | 0.73 | 0.12 | 0.69 | -0.14 | 1.36 | 0.47 |
| BAIAP2L2 | 0.02 | -0.30 | 0.51 | -0.02 | 0.19 | -0.39 | 0.72 | 0.22 |
| CD34 | 0.80 | -0.04 | 2.75 | 1.35 | 0.89 | 0.00 | 2.47 | 1.64 |
| FRMD3 | -0.04 | 0.01 | 0.82 | 0.12 | 0.26 | 0.05 | 0.65 | -0.01 |
| MEG3 | -0.27 | -0.82 | 0.49 | 0.15 | -0.04 | -0.36 | 0.94 | 0.66 |
| PLAUR | -0.52 | -0.92 | 1.42 | 1.02 | 0.28 | 0.22 | 1.10 | 0.41 |
| APOBEC3F | 0.33 | -0.52 | 0.62 | -0.03 | 0.03 | -0.30 | 0.74 | 0.69 |
| GFOD1 | -0.25 | -0.13 | 0.92 | 0.41 | -0.01 | 0.01 | 0.88 | 0.33 |
| CLCA1 | 0.11 | -1.07 | 1.58 | -0.31 | 0.29 | -0.87 | 1.12 | -0.72 |
| FZD7 | -0.43 | -0.01 | 0.78 | 0.63 | -0.93 | 0.01 | 1.07 | 0.83 |
| ATP2A3 | 0.02 | -0.70 | 1.15 | 0.58 | -0.40 | -0.99 | 0.53 | -0.01 |
| ATP2A3 | -0.11 | -0.50 | 1.24 | 0.21 | 0.29 | -0.30 | 0.68 | -0.02 |
| C1orf24 | -0.67 | -0.23 | 0.94 | 0.69 | -0.19 | -0.40 | 0.92 | 0.64 |
| PRKCB1 | 0.52 | 0.43 | 1.46 | 1.17 | -0.20 | -0.18 | 0.56 | -0.07 |
| RASGRP2 | -0.22 | -0.25 | 0.73 | 0.46 | 0.26 | 0.33 | 0.49 | 0.41 |
| FAM83A | -0.60 | -0.36 | 1.43 | 0.82 | -0.17 | -0.47 | 0.83 | 0.66 |
| ANTXR2 | -0.14 | 0.28 | 1.09 | 0.22 | 0.18 | 0.02 | 1.02 | 0.48 |
| GPR56 | -0.06 | -1.18 | 2.13 | 0.63 | 0.39 | -0.75 | 1.93 | 0.63 |
| CENTD2 | 0.13 | -0.09 | 0.58 | 0.24 | 0.23 | 0.08 | 0.74 | 0.43 |
| DPY19L4 | 0.04 | -0.96 | 0.54 | -0.04 | 0.25 | -0.14 | 0.89 | 0.48 |
| AK1 | -0.01 | -0.67 | 0.58 | 0.10 | 0.01 | -0.78 | 0.36 | -0.27 |
| SCPEP1 | -0.02 | -0.97 | 0.59 | 0.01 | 0.13 | -0.56 | 0.68 | -0.02 |
| DHRS9 | 0.31 | -0.47 | 2.90 | 1.11 | 0.14 | 0.29 | 1.46 | 0.52 |
| LST1 | 0.27 | -1.02 | 2.48 | 1.00 | 0.04 | -0.66 | 1.71 | 0.32 |
| C1orf116 | -0.51 | -0.57 | 0.63 | -0.34 | 0.32 | -0.84 | 0.86 | 0.34 |
| SLC16A10 | 0.00 | -0.40 | 0.98 | 0.57 | 0.00 | -0.24 | 0.72 | 0.29 |
| FILIP1L | -0.03 | -0.25 | 0.66 | -0.02 | -0.35 | 0.41 | 0.66 | 0.03 |
| GSDMB | -0.44 | -0.68 | 0.45 | 0.37 | -0.07 | -0.67 | 0.76 | 0.46 |
| EDEM1 | 0.45 | -0.29 | 0.68 | 0.27 | -0.02 | -0.51 | 0.49 | 0.02 |
| LOC728323 | -0.54 | -0.47 | 1.23 | 0.44 | 0.17 | -0.34 | 0.88 | 0.21 |
| PIB5PA | -0.13 | -0.74 | 0.70 | 0.10 | 0.20 | -0.32 | 0.65 | 0.34 |
| DUSP6 | 0.13 | 0.01 | 1.59 | 0.38 | -0.01 | -0.42 | 0.48 | -0.32 |
| LOC389791 | -0.48 | -0.76 | 0.36 | -0.37 | 0.42 | -0.33 | 0.84 | 0.37 |
| CD24 | -0.01 | -0.02 | 1.91 | 1.09 | 0.01 | -0.09 | 1.78 | 0.96 |
| CD24 | -0.46 | -0.35 | 1.19 | 0.78 | -0.10 | -0.33 | 1.12 | 0.64 |
