# Supplementary material for: Functional Characterization of D9, a Novel Deazaneplanocin A (DZNep) Analog, in Targeting Acute Myeloid Leukemia (AML)
Source: PLoS One. 2015 Apr 30;10(4):e0122983. doi: 10.1371/journal.pone.0122983 (PMC4415792; doi:10.1371/journal.pone.0122983)
Supplement: S8 Table — Table showing the averaged values of 46 probes of Integrin members. (DOCX) [file pone.0122983.s008.docx]

**S8 Table. Normalized microarray data of Integrins**

| **ProbeID** | **Symbol** | **DMSO** | **D9** | **Ara-C** | **D9+Ara-C** |
| --- | --- | --- | --- | --- | --- |
| 3390424 | ITGAD | 0.01 | 0.01 | -0.01 | -0.01 |
| 5720075 | ITGA8 | 0.27 | -3.84 | 0.33 | 1.68 |
| 5390201 | ITGA6 | 2.15 | -2.48 | -0.08 | 1.59 |
| 10433 | ITGA10 | -0.35 | -0.07 | -0.05 | 0.05 |
| 520670 | ITGA11 | -0.86 | 3.10 | 5.96 | 2.67 |
| 1710070 | ITGAM | 0.55 | -0.27 | 2.11 | 1.29 |
| 1770561 | ITGA11 | 0.03 | -0.94 | 1.07 | 0.46 |
| 1710070 | ITGAM | 0.55 | -0.27 | 2.11 | 1.29 |
| 6330338 | ITGA5 | -0.28 | -0.45 | 1.00 | 0.32 |
| 460220 | ITGA1 | 0.01 | 0.01 | -0.01 | -0.01 |
| 2140678 | ITGA6 | 0.00 | 0.00 | 5.09 | -0.01 |
| 7570608 | ITGA6 | 0.01 | 0.01 | -0.01 | -0.01 |
| 5340468 | ITGA2B | 0.06 | -0.56 | 0.99 | 0.27 |
| 3890541 | ITGA2 | -0.27 | -1.35 | -0.49 | 0.72 |
| 4180494 | ITGAL | -0.01 | 3.15 | 0.36 | 4.34 |
| 2000368 | ITGA3 | 0.61 | 0.46 | 0.83 | -0.73 |
| 1430196 | ITGA3 | 0.00 | 4.17 | -0.01 | -0.02 |
| 4490500 | ITGAX | -0.05 | -0.05 | 8.24 | 5.03 |
| 4250315 | ITGA9 | -0.39 | -0.44 | 0.35 | 0.03 |
| 7200768 | ITGA4 | -0.47 | 0.18 | -0.41 | 0.12 |
| 4120341 | ITGAE | 0.08 | 0.20 | 0.01 | -0.14 |
| 7040592 | ITGAX | 0.01 | 0.01 | -0.01 | -0.01 |
| 2690209 | ITGAV | -0.01 | 0.01 | 0.54 | 0.19 |
| 3400537 | ITGA7 | -0.29 | -0.21 | 0.15 | -0.13 |
| 1570598 | ITGB4 | 0.00 | 0.00 | 3.14 | -0.01 |
| 3990379 | ITGB7 | -0.68 | -0.57 | 0.97 | 0.65 |
| 6380142 | ITGB5 | -0.43 | -0.24 | -3.48 | -3.48 |
| 7200156 | ITGB2 | -0.40 | -0.53 | 0.29 | 0.10 |
| 3940132 | ITGB4 | 0.00 | 0.00 | 7.18 | 5.41 |
| 4490367 | ITGB4 | -1.37 | -0.84 | -0.03 | -1.39 |
| 610452 | ITGB1 | 0.08 | -0.06 | 0.24 | -0.18 |
| 6550379 | ITGB4 | 0.01 | 0.01 | -0.01 | -0.01 |
| 110440 | ITGB1 | 0.00 | -0.20 | 0.27 | -0.24 |
| 5910139 | ITGB1 | 0.01 | 0.01 | -0.01 | -0.01 |
| 3450301 | ITGB5 | 2.43 | 1.76 | 1.60 | -0.20 |
| 4040491 | ITGB1 | 0.01 | 0.01 | -0.01 | -0.01 |
| 650437 | ITGB6 | 0.01 | 0.01 | -0.01 | -0.01 |
| 1470685 | ITGB3 | 0.16 | -1.94 | 2.08 | 0.73 |
| 2070296 | ITGB1 | -0.64 | -0.21 | -0.04 | -1.10 |
| 3890373 | ITGB2 | -0.44 | -0.46 | 0.50 | 0.18 |
| 2690689 | ITGB8 | 0.01 | 0.01 | -0.01 | -0.01 |
| 5360243 | ITGB5 | 0.52 | -2.96 | 0.82 | 1.09 |
| 5360243 | ITGB5 | 0.52 | -2.96 | 0.82 | 1.09 |
| 2490411 | ITGB5 | 0.11 | -0.61 | 1.20 | 0.05 |
| 2650114 | ITGB5 | -0.08 | -0.79 | 0.73 | 0.06 |
| 650066 | ITGB1 | 0.05 | -0.13 | 0.38 | 0.18 |
| **AVE** |  | **0.03** | **-0.22** | **0.97** | **0.48** |
