# Supplementary material for: Functional Characterization of D9, a Novel Deazaneplanocin A (DZNep) Analog, in Targeting Acute Myeloid Leukemia (AML)
Source: PLoS One. 2015 Apr 30;10(4):e0122983. doi: 10.1371/journal.pone.0122983 (PMC4415792; doi:10.1371/journal.pone.0122983)
Supplement: S9 Table — Table showing the averaged values of 24 probes of Laminins. (DOCX) [file pone.0122983.s009.docx]

**S9 Table. Normalized microarray data of Laminins**

| **ProbeID** | **Symbol** | **DMSO** | **D9** | **Ara-C** | **D9+Ara-C** |
| --- | --- | --- | --- | --- | --- |
| 2190113 | LAMA1 | 0.01 | 0.01 | -0.01 | -0.01 |
| 5360110 | LAMA1 | 0.01 | 0.01 | -0.01 | -0.01 |
| 610037 | LAMA1 | 0.22 | 0.42 | 0.25 | -0.07 |
| 1470296 | LAMA2 | 0.00 | 0.00 | 2.93 | -0.01 |
| 360181 | LAMA2 | 0.00 | 0.00 | 3.40 | -0.02 |
| 2650612 | LAMA3 | 0.71 | -1.16 | -0.01 | 0.68 |
| 6480592 | LAMA3 | 0.01 | 0.01 | -0.01 | -0.01 |
| 4120358 | LAMA3 | -3.91 | 0.65 | -1.13 | 0.95 |
| 4670553 | LAMA3 | -1.48 | -0.64 | 1.10 | 0.08 |
| 7160253 | LAMA4 | 3.75 | -0.01 | -0.02 | 2.05 |
| 5130435 | LAMA4 | 0.01 | 0.01 | -0.01 | -0.01 |
| 4040176 | LAMA5 | -0.04 | -0.48 | 0.28 | -0.09 |
| 1510392 | LAMB1 | 0.49 | -0.34 | 1.13 | 1.01 |
| 2630746 | LAMB1 | -0.88 | -1.21 | -0.01 | 0.28 |
| 7650189 | LAMB2 | -0.20 | 0.45 | 1.73 | 1.98 |
| 730040 | LAMB3 | 0.14 | -0.03 | 1.33 | 0.23 |
| 6350243 | LAMB3 | -1.48 | -1.48 | 2.04 | -1.49 |
| 4590739 | LAMB3 | 0.01 | 0.01 | -0.01 | -0.01 |
| 1710669 | LAMB4 | 0.01 | 0.01 | -0.01 | -0.01 |
| 4120086 | LAMC1 | 0.71 | -0.07 | 0.03 | -0.03 |
| 4390100 | LAMC2 | 0.01 | 0.01 | -0.01 | -0.01 |
| 2230594 | LAMC2 | 0.01 | 0.01 | -0.01 | -0.01 |
| 4150725 | LAMC2 | 0.00 | 3.13 | -0.01 | -0.01 |
| 6280541 | LAMC3 | -0.15 | -0.76 | 0.10 | -0.08 |
| **AVE** |  | **-0.09** | **-0.06** | **0.55** | **0.22** |
