# Supplementary material for: Functional Characterization of D9, a Novel Deazaneplanocin A (DZNep) Analog, in Targeting Acute Myeloid Leukemia (AML)
Source: PLoS One. 2015 Apr 30;10(4):e0122983. doi: 10.1371/journal.pone.0122983 (PMC4415792; doi:10.1371/journal.pone.0122983)
Supplement: S10 Table — Table showing the averaged values of 8 probes of cytokines. (DOCX) [file pone.0122983.s010.docx]

**S10 Table. Normalized microarray data of cytokines**

| **ProbeID** | **Symbol** | **DMSO** | **D9** | **Ara-C** | **D9+Ara-C** |
| --- | --- | --- | --- | --- | --- |
| 6280332 | CXCL16 | -0.70 | 0.13 | 2.80 | 1.36 |
| 540377 | CXCL5 | 0.64 | 0.17 | 1.76 | 1.05 |
| 4670390 | CXCL2 | -0.09 | 0.03 | 1.66 | 0.10 |
| 5360048 | CCL23 | 0.00 | 0.01 | 5.15 | -0.01 |
| 6110343 | CCL23 | -0.10 | -1.33 | 3.14 | 0.82 |
| 1030333 | CCL2 | 0.00 | 0.00 | 6.33 | 6.30 |
| 7570408 | CCL5 | -0.44 | -0.17 | 2.19 | 0.75 |
| 620717 | CCL5 | -0.26 | -1.46 | 2.31 | 0.30 |
| **AVE** |  | **-0.12** | **-0.33** | **3.17** | **1.33** |
