# Supplementary material for: Functional Characterization of D9, a Novel Deazaneplanocin A (DZNep) Analog, in Targeting Acute Myeloid Leukemia (AML)
Source: PLoS One. 2015 Apr 30;10(4):e0122983. doi: 10.1371/journal.pone.0122983 (PMC4415792; doi:10.1371/journal.pone.0122983)
Supplement: S11 Table — Table showing 8 probes of receptors of cytokines extracted from normalized microarray data of CD34+CD38- double-selected TF-1a cells treated as indicated. (DOCX) [file pone.0122983.s011.docx]

**S11 Table. Normalized microarray data of the receptors of cytokines**

| **ProbeID** | **Symbol** | **DMSO** | **D9** | **Ara-C** | **D9+Ara-C** |
| --- | --- | --- | --- | --- | --- |
| 3890400 | CXCR5 | -1.79 | -0.32 | 1.06 | -3.84 |
| 7320176 | CCR1 | 0.88 | -0.96 | 2.15 | -1.47 |
| 7570154 | CCR4 | -0.39 | -1.89 | 2.11 | 0.43 |
| 5360484 | CCR9 | 0.01 | 0.01 | -0.01 | -0.01 |
| 2190671 | CCR3 | -2.48 | -4.73 | 1.88 | 1.31 |
| 5390246 | CCR7 | -2.58 | -1.54 | 1.39 | 0.81 |
| 3360372 | CCRL2 | -0.19 | -0.39 | 1.03 | 0.39 |
| 2070168 | CX3CR1 | -0.05 | -0.05 | 6.06 | 1.49 |
| **AVE** |  | **-0.82** | **-1.23** | **1.96** | **-0.11** |
